# Supplementary material for: Vascular and metabolic risk factor differences prior to dementia diagnosis: a multidatabase case–control study using European electronic health records
Source: BMJ Open. 2020 Nov 14;10(11):e038753. doi: 10.1136/bmjopen-2020-038753 (PMC7668358; doi:10.1136/bmjopen-2020-038753)
Supplement: Supplementary data [file bmjopen-2020-038753supp001.pdf]

Supplementary Table 1: Vascular risk factor status at each measurement period by database

Risk factors	Case/ Control status	AUH					
		10- 12 years	8- 10 years	6- 8 years	4- 6 years	2- 4 years	0- 2 Years*
Mean BMI (SD)	Case				26.03 (4.36)	24.96 (4.74)	24.27 (4.56)
	Control				25.40 (4.49)	25.56 (4.46)	25.38 (4.61)
Mean Total cholesterol (SD)	Case	5.75 (1.10)	5.44 (1.03)	5.27 (1.07)	5.11 (1.06)	4.99 (1.09)	4.86 (1.10)
	Control	5.72 (1.04)	5.46 (1.07)	5.26 (1.08)	5.12 (1.05)	5.00 (1.05)	4.87 (1.06)
		HSD					
Risk factors	Case/ Control status						
		10- 12 years	8- 10 years	6- 8 years	4- 6 years	2- 4 years	0- 2 Years
Mean BMI (SD)	Case	27.33 (4.59)	27.59 (4.76)	27.29 (4.72)	26.98 (4.79)	26.71 (4.87)	26.40 (4.89)
	Control	27.35 (4.39)	27.46 (4.25)	27.44 (4.25)	27.27 (4.21)	27.07 (4.25)	26.90 (4.38)
Mean SBP (SD)	Case	143.84 (15.78)	142.95 (15.47)	141.99 (15.28)	140.72 (15.39)	139.44 (15.38)	137.22 (15.64)
	Control	144.95 (15.49)	144.11 (15.23)	143.07 (15.26)	141.95 (15.12)	140.65 (14.93)	139.03 (14.69)
Mean DBP (SD)	Case	82.97 (7.70)	82.17 (7.42)	81.34 (7.40)	80.55 (7.36)	79.72 (7.31)	78.83 (7.38)
	Control	81.93 (7.56)	81.48 (7.42)	80.59 (7.30)	79.89 (7.29)	79.16 (7.45)	78.24 (7.71)
Mean Total cholesterol (SD)	Case	5.95 (0.98)	5.85 (0.99)	5.73 (1.01)	5.63 (1.01)	5.51 (1.02)	5.39 (1.04)
	Control	5.93 (1.02)	5.83 (1.03)	5.70 (1.05)	5.59 (1.06)	5.47 (1.08)	5.31 (1.10)
		IPCI					
Risk factors	Case/ Control status						
		10- 12 years	8- 10 years	6- 8 years	4- 6 years	2- 4 years	0- 2 Years
Mean BMI (SD)	Case	27.78 (4.64)	27.48 (4.62)	27.26 (4.45)	27.11 (4.37)	26.80 (4.41)	26.25 (4.54)
	Control	28.01 (4.64)	27.83 (4.74)	27.62 (4.74)	27.57 (4.63)	27.54 (4.61)	27.32 (4.54)
Mean SBP (SD)	Case	150.35 (17.94)	149.65 (17.80)	147.98 (17.74)	146.22 (17.24)	144.34 (17.39)	141.25 (17.75)

	Control	150.86 (17.86)	150.08 (17.25)	148.63 (17.03)	147.31 (16.94)	145.88 (16.90)	144.28 (16.89)
Mean DBP (SD)	Case	83.68 (8.40)	82.75 (8.39)	81.41 (8.26)	80.23 (8.53)	79.02 (8.61)	77.75 (8.81)
	Control	84.18 (8.16)	82.88 (8.28)	81.59 (8.40)	80.52 (8.41)	79.47 (8.48)	78.45 (8.48)
Mean Total cholesterol (SD)	Case	5.70 (1.11)	5.52 (1.11)	5.34 (1.17)	5.23 (1.16)	5.12 (1.15)	5.01 (1.15)
	Control	5.73 (1.01)	5.48 (1.13)	5.33 (1.17)	5.24 (1.19)	5.18 (1.14)	5.07 (1.12)

		**SIDIAP**					
Risk factors	Case/ Control status	10- 12 years	8- 10 years	6- 8 years	4- 6 years	2- 4 years	0- 2 Years
Mean BMI (SD)	Case		29.10 (4.39)	28.79 (4.47)	28.49 (4.45)	28.11 (4.49)	27.54 (4.55)
	Control		29.74 (4.53)	29.49 (4.54)	29.23 (4.56)	28.97 (4.61)	28.67 (4.68)
Mean SBP (SD)	Case		137.73 (15.19)	137.33 (14.54)	136.95 (14.36)	136.10 (14.18)	134.19 (14.33)
	Control		138.56 (14.24)	138.23 (14.08)	137.73 (13.78)	136.96 (13.43)	135.90 (13.03)
Mean DBP (SD)	Case		76.49 (8.18)	75.75 (7.88)	75.02 (7.95)	74.21 (8.00)	73.15 (8.17)
	Control		77.12 (7.90)	76.47 (7.91)	75.51 (7.90)	74.56 (7.98)	73.49 (7.99)
Mean Total cholesterol (SD)	Case		5.39 (0.93)	5.35 (0.93)	5.30 (0.94)	5.22 (0.96)	5.10 (0.99)
	Control		5.43 (0.93)	5.38 (0.93)	5.32 (0.94)	5.26 (0.97)	5.16 (0.98)

		**THIN**					
Risk factors	Case/ Control status	10- 12 years	8- 10 years	6- 8 years	4- 6 years	2- 4 years	0- 2 Years
Mean BMI (SD)	Case	26.74 (4.77)	26.66 (4.83)	26.47 (4.87)	26.2 (8.90)	25.75 (5.03)	24.95 (5.19)
	Control	26.57 (4.49)	26.57 (4.54)	26.53 (4.60)	26.41 (4.68)	26.32 (4.75)	26.18 (4.89)
Mean SBP (SD)	Case	146.09 (19.46)	145.11 (19.00)	144.23 (18.32)	142.98 (17.96)	140.59 (17.84)	136.75 (17.34)
	Control	148.22 (18.33)	147.47 (17.80)	146.46 (17.44)	145.04 (17.04)	143.35 (16.74)	141.29 (16.61)
Mean DBP (SD)	Case	81.39 (9.69)	80.36 (8.64)	79.44 (9.37)	78.47 (9.30)	77.21 (9.28)	75.72 (9.13)
	Control	81.05 (8.88)	80.00 (8.73)	78.97 (8.69)	77.93 (8.54)	76.99 (8.43)	75.93 (8.42)
Mean Total cholesterol (SD)	Case	5.20 (1.52)	5.07 (1.38)	4.98 (1.30)	4.89 (1.25)	4.82 (1.22)	4.75 (1.19)

	Control	5.32 (1.31)	5.05 (1.31)	4.90 (1.28)	4.83 (1.24)	4.79 (1.20)	4.73 (1.18)

Risk factors	Case/ Control status	**IMIM-UPF**					
		10- 12 years	8- 10 years	6- 8 years	4- 6 years	2- 4 years	0- 2 Years
Mean BMI (SD)	Case		28.69 (4.50)	29.33 (5.13)	29.01 (4.92)	27.06 (4.43)	26.88 (4.63)
	Control		30.35 (4.70)	29.28 (4.86)	28.85 (4.49)	28.42 (4.58)	27.92 (4.51)
Mean Total cholesterol (SD)	Case	5.31 (1.09)	5.26 (1.09)	5.14 (1.15)	5.01 (1.17)	4.84 (1.15)	4.27 (1.08)
	Control	5.50 (1.07)	5.35 (1.10)	5.18 (1.12)	5.01 (1.12)	4.86 (1.13)	4.71 (1.12)

*Time periods (years prior to index date)

Supplementary Table 2: Proportions of patients with missing data for each vascular risk factor in each database

All databases		10- 12	8- 10	6- 8	4- 6	2- 4	0-2*
BMI	Cases	91%	85%	77%	69%	61%	52%
	Control	87%	81%	75%	67%	60%	56%
SBP	Cases	78%	70%	59%	47%	34%	19%
	Control	70%	62%	51%	40%	29%	22%
DBP	Cases	78%	70%	59%	47%	34%	19%
	Control	70%	62%	51%	40%	29%	22%
Total Cholesterol	Cases	92%	85%	75%	64%	53%	39%
	Control	89%	82%	72%	61%	51%	43%

AUH		10- 12	8- 10	6- 8	4- 6	2- 4	0-2*
BMI	Cases				99%	87%	50%
	Control				98%	84%	62%
Total Cholesterol	Cases	78%	65%	55%	44%	38%	23%
	Control	79%	68%	55%	44%	35%	29%

HSD		10- 12	8- 10	6- 8	4- 6	2- 4	0-2*
BMI	Cases	94%	91%	88%	85%	82%	78%
	Control	94%	91%	88%	85%	82%	79%
SBP	Cases	79%	71%	64%	57%	52%	47%
	Control	80%	72%	64%	57%	52%	49%
DBP	Cases	79%	71%	63%	57%	52%	47%
	Control	80%	72%	64%	57%	52%	49%
Total Cholesterol	Cases	83%	77%	70%	64%	60%	54%
	Control	82%	76%	69%	63%	57%	53%

IPCI		10- 12	8- 10	6- 8	4- 6	2- 4	0-2*
BMI	Cases	93%	88%	83%	76%	72%	67%
	Control	92%	88%	82%	75%	70%	65%
SBP	Cases	59%	53%	45%	36%	29%	18%
	Control	54%	48%	41%	33%	27%	21%
DBP	Cases	60%	53%	45%	36%	29%	18%
	Control	55%	48%	41%	41%	27%	21%
Total Cholesterol	Cases	83%	77%	70%	63%	58%	55%
	Control	83%	77%	67%	48%	53%	50%

SIDIAP		10- 12	8- 10	6- 8	4- 6	2- 4	0-2*
BMI	Cases		93%	80%	67%	54%	45%
	Control		93%	82%	68%	55%	46%
SBP	Cases		90%	74%	54%	35%	20%
	Control		90%	75%	56%	36%	24%
DBP	Cases		90%	74%	54%	54%	20%
	Control		90%	75%	56%	99%	24%
Total Cholesterol	Cases		92%	77%	60%	41%	24%
	Control		93%	78%	61%	44%	32%

THIN		10- 12	8- 10	6- 8	4- 6	2- 4	0-2*
BMI	Cases	83%	77%	72%	67%	60%	52%
	Control	74%	69%	65%	60%	58%	57%
SBP	Cases	68%	60%	52%	44%	34%	16%
	Control	50%	42%	35%	28%	22%	18%
DBP	Cases	68%	60%	52%	44%	34%	16%
	Control	50%	42%	35%	28%	22%	18%
Total Cholesterol	Cases	88%	82%	75%	69%	61%	48%
	Control	83%	76%	68%	61%	55%	50%

IMIM-UPF		10- 12	8- 10	6- 8	4- 6	2- 4	0-2*
BMI	Cases		99%	99%	98%	98%	94%
	Control		99%	99%	98%	97%	95%
Total Cholesterol	Cases	86%	81%	76%	70%	64%	31%
	Control	87%	83%	79%	75%	71%	68%

*Time periods (years prior to index date)

Supplementary Table 3: Meta-analysed mean number of missing data points* (95% confidence intervals)

	BMI		SBP		DBP		Total cholesterol	
**Variable**	**Control**	**Case**	**Control**	**Case**	**Control**	**Case**	**Control**	**Case**
**Age at index date**								
Under 60	2.75 (1.74, 3.76)	2.54 (1.13, 3.95)	2.78 (1.77, 3.79)	2.77 (1.68, 3.86)	2.78 (1.77, 3.79)	2.77 (1.68, 3.86)	2.67 (1.42, 3.92)	2.58 (1.46, 3.70)
60- 69	2.81 (1.74, 3.88)	2.76 (1.28, 4.24)	2.75 (1.77, 3.73)	2.73 (1.50, 3.96)	2.75 (1.77, 3.73)	2.73 (1.50, 3.96)	2.89 (1.43, 4.35)	2.29 (0.84, 3.74)
70- 79	2.89 (1.83, 3.95)	2.84 (1.02, 4.66)	2.81 (1.63, 3.99)	2.85 (1.56, 4.14)	2.81 (1.63, 3.99)	2.85 (1.56, 4.14)	2.28 (1.27, 3.29)	2.44 (1.15, 3.73)
80- 89	2.81 (1.25, 4.37)	2.86 (1.3, 4.42)	2.17 (0.85, 3.49)	2.67 (1.51, 3.83)	2.17 (0.85, 3.49)	2.67 (1.51, 3.83)	2.76 (1.55, 3.97)	2.54 (1.22, 3.86)
90 and over	2.54 (1.13, 3.95)	2.65 (1.16, 4.14)	2.71 (1.26, 4.16)	2.45 (1.16, 3.74)	2.71 (1.26, 4.16)	2.45 (1.16, 3.74)	2.44 (1.10, 3.78)	2.77 (1.42, 4.12)
**Gender**								
Male	2.78 (1.19, 4.37)	2.71 (1.08, 4.34)	2.60 (1.51, 3.69)	2.74 (1.69, 3.79)	2.60 (1.51, 3.69)	2.74 (1.69, 3.79)	2.24 (1.23, 3.25)	2.56 (1.47, 3.65)
Female	2.71 (1.28, 4.14)	2.75 (1.17, 4.33)	2.69 (1.37, 4.01)	2.64 (1.38, 3.90)	2.69 (1.37, 4.01)	2.64 (1.38, 3.90)	2.32 (1.09, 3.55)	2.48 (1.34, 3.62)
**Co-morbidities**								
AMI	2.55 (1.47, 3.63)	2.63 (1.38, 3.88)	3.01 (1.94, 4.08)	2.97 (1.85, 4.09)	3.01 (1.94, 4.08)	2.97 (1.85, 4.09)	2.12 (0.88, 3.36)	2.10 (0.64, 3.56)
DM	2.76 (1.62, 3.90)	2.71 (1.35, 4.07)	2.70 (1.45, 3.95)	2.75 (1.63, 3.87)	2.70 (1.45, 3.95)	2.75 (1.63, 3.87)	2.07 (0.62, 3.52)	2.13 (0.77, 3.49)

Stroke	2.82 (1.59, 4.05)	2.78 (1.32, 4.24)	2.81 (1.36, 4.26)	2.72 (1.49, 3.95)	2.81 (1.36, 4.26)	2.72 (1.49, 3.95)	2.87 (1.01, 4.73)	2.39 (0.72, 4.06)
Hypertension	2.12 (1.11, 3.13)	2.05 (0.80, 3.30)	2.43 (1.32, 3.54)	2.39 (1.38, 3.40)	2.43 (1.32, 3.54)	2.39 (1.38, 3.40)	2.19 (0.78, 3.60)	2.14 (0.55, 3.73)
Total sample	2.75	2.73	2.67	2.66	2.67	2.66	2.30	2.50

*From a denominator of 6 data points apart from the following: UPF-BMI 5 data points; AUH-BMI 3 data points; SIDIAP variables 5 data points

AMI: acute myocardial infarction; DM: type 2 diabetes mellitus
