# Supplementary material for: Vascular and metabolic risk factor differences prior to dementia diagnosis: a multidatabase case–control study using European electronic health records
Source: BMJ Open. 2020 Nov 14;10(11):e038753. doi: 10.1136/bmjopen-2020-038753 (PMC7668358; doi:10.1136/bmjopen-2020-038753)
Supplement: Author's manuscript [file bmjopen-2020-038753.draft_revisions.pdf]

BMJ Open is committed to open peer review. As part of this commitment we make the peer review history of every article we publish publicly available.

When an article is published we post the peer reviewers' comments and the authors' responses online. We also post the versions of the paper that were used during peer review. These are the versions that the peer review comments apply to.

The versions of the paper that follow are the versions that were submitted during the peer review process. They are not the versions of record or the final published versions. They should not be cited or distributed as the published version of this manuscript.

BMJ Open is an open access journal and the full, final, typeset and author-corrected version of record of the manuscript is available on our site with no access controls, subscription charges or pay-per-view fees (http://bmjopen.bmj.com).

If you have any questions on BMJ Open's open peer review process please email info.bmjopen@bmj.com

BMJ Open

# Vascular and metabolic risk factor differences prior to dementia diagnosis: a multi-database case control study using European electronic health records

| | |
|---|---|
| Journal: | *BMJ Open* |
| Manuscript ID | bmjopen-2020-038753 |
| Article Type: | Original research |
| Date Submitted by the Author: | 14-Apr-2020 |
| Complete List of Authors: | Perera, Gayan; Institute of Psychiatry Psychology and Neuroscience, Psychological Medicine<br>Rijnbeek, P; Erasmus Medical Center<br>Alexander, Myriam; GlaxoSmithKline Plc, Quantitative Sciences, , Uxbridge, United Kingdom.<br>Ansell, David; University of Birmingham, Institute of Applied Health Research<br>Avillach, Paul; Harvard Medical School<br>Duarte-Salles, Talita; IDIAP Jordi Gol<br>Gordon, Mark ; Teva Pharmaceuticals USA Inc, Specialty Clinical Development, Neurology and Psychiatry<br>Lapi, Francesco; Italian College of General Practitioners and Primary Care<br>Mayer, Miguel; Hospital del Mar Institute for Medical Research<br>Pasqua, Alessandro; Health Search, Italian College of General Practitioners and Primary Care<br>Pedersen, Lars; Aarhus University Hospital, Department of Clinical Epidemiology<br>van Der Lei, Johan; Erasmus Medical Center<br>Visser, Pieter; Maastricht University, Department of Psychiatry and Neuropsychology<br>Stewart, Robert; Institute of Psychiatry Psychology and Neuroscience, Psychological Medicine |
| Keywords: | Dementia < NEUROLOGY, Physiology < BASIC SCIENCES, Health informatics < BIOTECHNOLOGY & BIOINFORMATICS, Hypertension < CARDIOLOGY, EPIDEMIOLOGY, MENTAL HEALTH |
| | |

SCHOLARONE™
Manuscripts

*I, the Submitting Author has the right to grant and does grant on behalf of all authors of the Work (as defined in the below author licence), an exclusive licence and/or a non-exclusive licence for contributions from authors who are: i) UK Crown employees; ii) where BMJ has agreed a CC-BY licence shall apply, and/or iii) in accordance with the terms applicable for US Federal Government officers or employees acting as part of their official duties; on a worldwide, perpetual, irrevocable, royalty-free basis to BMJ Publishing Group Ltd ("BMJ") its licensees and where the relevant Journal is co-owned by BMJ to the co-owners of the Journal, to publish the Work in this journal and any other BMJ products and to exploit all rights, as set out in our licence.*

*The Submitting Author accepts and understands that any supply made under these terms is made by BMJ to the Submitting Author unless you are acting as an employee on behalf of your employer or a postgraduate student of an affiliated institution which is paying any applicable article publishing charge ("APC") for Open Access articles. Where the Submitting Author wishes to make the Work available on an Open Access basis (and intends to pay the relevant APC), the terms of reuse of such Open Access shall be governed by a Creative Commons licence – details of these licences and which Creative Commons licence will apply to this Work are set out in our licence referred to above.*

*Other than as permitted in any relevant BMJ Author's Self Archiving Policies, I confirm this Work has not been accepted for publication elsewhere, is not being considered for publication elsewhere and does not duplicate material already published. I confirm all authors consent to publication of this Work and authorise the granting of this licence.*

For peer review only

# Vascular and metabolic risk factor differences prior to dementia diagnosis: a multi-database case control study using European electronic health records

Gayan Perera[1], PhD; Peter Rijnbeek[2], PhD; Myriam Alexander[3], PhD; David Ansell[4], MD PhD; Paul Avillach[5,6], MD, PhD; Talita Duarte-Salles, MPH, PhD[7]; Mark Forrest Gordon[8], MD; Francesco Lapi[9], PhD; Miguel A. Mayer[10], MD, PhD; Alessandro Pasqua[9], MSc; Lars Pedersen[11], PhD; Johan van der Lei[2], PhD, MD; Pieter Jelle Visser[12,13,14], MD PhD; Robert Stewart[1,15], MD, FRCPsych.

[1]King's College London (Institute of Psychiatry, Psychology and Neuroscience), London, United Kingdom.

[2]Department of Medical Informatics, Erasmus Medical Center, Rotterdam, Netherlands

[3]Quantitative Sciences, GlaxoSmithKline, Uxbridge, United Kingdom.

[4]Institute of Applied Health Research, University of Birmingham, Birmingham, UK

[5]Aarhus University, Aarhus, Denmark

[6]Department of Biomedical Informatics, Harvard Medical School, Boston, MA, USA

[7]Fundació Institut Universitari per a la recerca a l'Atenció Primària de Salut Jordi Gol i Gurina (IDIAPJGol), Barcelona, Spain

[8]Specialty Clinical Development, Neurology and Psychiatry, Teva Pharmaceuticals, Frazer, PA 19355 USA

[9]Health Search, Italian College of General Practitioners and Primary Care, Florence, Italy



For peer review only - http://bmjopen.bmj.com/site/about/guidelines.xhtml

[10]Universitat Pompeu Fabra and Hospital del Mar Medical Research Institute, Barcelona, Spain

[11]Department of Clinical Epidemiology, Aarhus University Hospital, Aarhus, Denmark

[12]Alzheimer Center Amsterdam, Department of Neurology, Amsterdam Neuroscience, Amsterdam UMC, Vrije Universiteit, Amsterdam, The Netherlands

[13]Department of Psychiatry and Neuropsychology, School for Mental Health and Neuroscience (MHeNS), Alzheimer Centrum Limburg, Maastricht University, Maastricht, The Netherlands

[14]Department of Neurobiology, Care Sciences and Society, Division of Neurogeriatrics, Karolinska Institutet, Stockholm Sweden.

[15]South London and Maudsley NHS Foundation Trust, London, United Kingdom

**Corresponding author:** Robert Stewart, Department of Psychological Medicine (Box 84), Institute of Psychiatry, Psychology and Neuroscience (King's College London), De Crespigny Park, London SE5 8AF, UK. Phone: +44 (0)20 7848 0778, Email: robert.stewart@kcl.ac.uk

**Funding statement:** The research leading to these results has received support from the Innovative Medicines Initiative (IMI) Joint Undertaking under EMIF grant agreement n° 115372, resources of which are composed of financial contribution from the European Union's Seventh Framework Programme (FP7/2007-2013) and EFPIA companies' in kind contribution. RS and GP are part-funded by the National Institute for Health Research



For peer review only - http://bmjopen.bmj.com/site/about/guidelines.xhtml

(NIHR) Biomedical Research Centre and Dementia Biomedical Research Unit at South London and Maudsley NHS Foundation Trust and King's College London.

**Competing interests statement:**

MA received funding from Roche and GSK as a contracted employee; GP received funding from Roche as contracted employee during this project; MFG has prior employment at Boehringer Ingelheim Pharmaceuticals, and is a current employee of Teva Pharmaceuticals; RS has received research funding from Roche, Janssen, GSK and Takeda.

**Authors' contributions:**

The analysis was conceived and designed by RS, PR and JVDL. Data extractions and advice on analysis design were provided by the data controllers (DA, TD-S, MAM, AP, LP, JVDL) in liaison with PR and PA. Analyses were carried out by LP and GP. The manuscript was written by GP and RS. Further comments and significant input were obtained from all remaining co-authors who also oversaw the planning and development of the project.

**Acknowledgements**

(see under funding, above)

**Data sharing statement:** No additional data are available. The datasets analysed during the current study are not publicly available due to confidentially of patients but are available via application to individual data custodians under the EMIF platform (http://www.emif.eu/).



For peer review only - http://bmjopen.bmj.com/site/about/guidelines.xhtml

**Licence statement:** This is an open access article distributed in accordance with the Creative Commons Attribution Non Commercial (CC BY-NC 4.0) license, which permits others to distribute, remix, adapt, build upon this work non-commercially, and license their derivative works on different terms, provided the original work is properly cited, appropriate credit is given, any changes made indicated, and the use is non-commercial. See: http://creativecommons.org/licenses/by-nc/4.0/.

For peer review only

**Manuscript word count:** 3020



For peer review only - http://bmjopen.bmj.com/site/about/guidelines.xhtml

**ABSTRACT**

OBJECTIVE: The objective of the study is to compare body mass index (BMI), systolic/diastolic blood pressure (SBP/DBP), and serum total cholesterol levels between dementia cases and controls at multiple time intervals prior to dementia onset, and to test time interval as a modifying factor for these associations.

DESIGN: Case control study.

SETTING: Six European electronic health records databases.

PARTICIPANTS: 291,780 cases at the date of first-recorded dementia diagnosis, compared to 29,170,549 controls randomly selected from the same databases, age- and sex-matched at this index date.

EXPOSURE: The following measures were extracted whenever recorded within each dataset: BMI (kg/m$^2$), SBP and DBP (mmHg), and serum total cholesterol (mmol/l). Levels for each of these variables were defined within six 2-year time intervals over the 12 years prior to the index date.

MAIN OUTCOMES: Case-control differences in exposures of interest were modelled for each time period and adjusted for demographic and clinical factors (ischaemic/unspecified stroke, type 2 diabetes mellitus, acute myocardial infarction, hypertension diagnosis, anti-hypertensive medication, cholesterol-lowering medication). Coefficients and interactions with time period were meta-analysed across the six databases.

RESULTS: Mean BMI (coefficient -1.16 kg/m2; 95% CI: -1.38, -0.93) and systolic BP (-2.83 mmHg; -4.49, -1.16) were lower in cases at diagnosis, and case-control differences were greater in more recent time periods, as indicated by significant case-x-time interaction and



For peer review only - http://bmjopen.bmj.com/site/about/guidelines.xhtml

case-x-time-squared interaction terms. Time variations in coefficients for cholesterol levels were less consistent between databases and those for diastolic BP were largely not significant.

DISCUSSION: Routine clinical data show emerging divergence in levels of BMI and SBP prior to the diagnosis of dementia but less evidence for DBP or total cholesterol levels. These divergences should receive at least some consideration in routine dementia risk screening, although underlying mechanisms still require further investigation.

Keywords: Dementia; blood pressure; body mass index; serum cholesterol; electronic health records; vascular risk factor trajectories.



For peer review only - http://bmjopen.bmj.com/site/about/guidelines.xhtml

**Strengths and limitations of this study**

1. We have investigated associations of blood pressure, body mass index and cholesterol levels with dementia and the time-dependency of such observations, using what we believe to be currently the largest confederation of health records data.

2. There are advantages in the generalisability of routine data and in patient populations who are less subject to participation selection than research cohorts.

3. The use of multiple data resources allowed the consistency of associations to be evaluated, as it is particularly important for clinical 'big data' where large sample sizes result in even very minor associations being identified as statistically significant, and where traditional heterogeneity statistics from meta-analyses are similarly uninformative.

4. The limitations of the study are those that typify routine administrative rather than research data, in that measurements were limited to those recorded in routine practice which could be harmonised across the six databases, and the stage of dementia at diagnosis was not known.

5. While the large samples will have reduced the impact of missing data on statistical power, potential bias arising from non-random missingness cannot be excluded, although we feel it is unlikely to account for the findings of interest.

For peer review only



For peer review only - http://bmjopen.bmj.com/site/about/guidelines.xhtml

## INTRODUCTION

Vascular risk factors are widely recognised as important in the aetiology of dementia; however, observed associations depend on the timing of measurement. For example, higher blood pressure (particularly systolic blood pressure; SBP) in mid-life is a predictor of dementia 10-20 years later [1,2,3], but people with dementia tend to have lower blood pressure at the time of the clinical onset than age-matched controls [1], accounted for by an exaggerated decline in systolic blood pressure prior to the onset of dementia [4,5,6]. Similar associations have been observed for body mass index (BMI), with mid-life obesity associated with increased risk of dementia [7,8], but dementia clinical onset associated with lower contemporaneous BMI [9] and preceded by accelerated weight loss [10]. Finally, higher mid-life cholesterol has been found to predict dementia in some studies [11], and a decline in cholesterol levels prior to dementia onset has been observed in others [12,13].

These findings have arisen almost exclusively from specifically recruited research cohorts followed over long periods – advantageous in many respects but potentially subject to selection, and therefore limited generalisability. The parameters of interest (BP, BMI, cholesterol levels) are routinely measured in clinical practice, but there has been no investigation of their associations with dementia and the time-dependency of such observations. We sought to pursue this in what we believe to be currently the largest confederation of health records data – that enabled by the European Medical Information Framework Innovative Medicines Initiative (EMIF-IMI) within which an Alzheimer's Disease (EMIF-AD) consortium was developed. The objective of the study reported here was to compare levels of the exposures of interest between dementia cases and controls at multiple time intervals prior to dementia onset, and to test time interval as a modifying factor for these associations. The specific hypothesis was that BMI, systolic and diastolic blood



For peer review only - http://bmjopen.bmj.com/site/about/guidelines.xhtml

pressure and serum total cholesterol would be lower in people with newly diagnosed dementia than in controls, and that these differences would have widened closer to the diagnosis date.

For peer review only



For peer review only - http://bmjopen.bmj.com/site/about/guidelines.xhtml

# METHODS

## Data resources

EMIF-IMI worked with several electronic health record (EHR) resources to render data available for analysis with appropriately robust data security and governance. EMIF datasets participating in a recent investigation of incidence and prevalence of diagnosed dementia were estimated to contain information on 182,776 people with a dementia diagnosis, with around 1,000,000 person-years of data prior to the diagnosis and 400,000 person-years after the diagnosis [14]. These resources included three primary care databases from the Netherlands, Italy and United Kingdom (Integrated Primary Care Information [IPCI], Health Search Database [HSD] and The Health Improvement Network [THIN] respectively) and two hospital catchment area databases in Jutland and Barcelona (Aarhus University Hospital Database [AUH] and Information System of Parc de Salut Mar [IMIM-UPF] respectively). In this study, the 'Information System for Research in Primary Care [SIDIAP], containing Catalan primary care records) was added as a new database. The final assembled data resource included a total 291,780 patients with a dementia diagnosis across the six databases.

This study created an age- and gender-matched case control study, comparing people with and without dementia on prior exposures of interest over the preceding 12 years, adjusting for relevant and available covariates, and investigating time interval as a modifying factor.

## Population

Cases were defined on the basis of a diagnosis of dementia (inclusion criterion) and the index date was set as that on which the first dementia diagnosis was recorded. The definition of dementia was extensively developed and harmonised across the EMIF-AD consortium,



For peer review only - http://bmjopen.bmj.com/site/about/guidelines.xhtml

taking into account the different coding systems used, as previously described in detail [14]. For each case, at the index date of first dementia diagnosis, 100 controls were drawn randomly from the same EHR, matched to the case on gender and age (+/- 2 years) at that index date, having excluded any dementia at or prior to that date. When there were not enough controls, one control was allowed to be used for several cases. Both cases and controls were restricted to people aged 50 years or over at the index date.

For peer review only

**Exposures**

The following measures were extracted whenever recorded within each dataset: BMI ($kg/m^2$), systolic BP (SBP; mmHg), diastolic BP (DBP; mmHg), and serum total cholesterol (mmol/l). BMI was extracted where recorded, or was calculated from recorded body weight and height data. Levels for each of these variables were defined within six 2-year time intervals over the 12 years prior to the index date, taking mean values if multiple measures were present for an individual within each interval. Percentages of missing data points were compared between cases and controls, and by age, gender and comorbidities.

**Covariates**

Data on age at index date and gender were extracted. In addition, the following binary variables were generated based on any recorded diagnosis/treatment prior to the index date: ischaemic/unspecified stroke, type 2 diabetes mellitus (diagnosis or antidiabetic medication), acute myocardial infarction, hypertension diagnosis, use of any anti-hypertensive medication and use of any cholesterol-lowering medication. Medication information was extracted from databases in which it was available from recorded prescriptions or dispensations; disorders



For peer review only - http://bmjopen.bmj.com/site/about/guidelines.xhtml

were defined according to pre-existing harmonised data extraction criteria used across the EMIF-IMI consortia [15].

**Statistical analysis**

The total pool of cases and controls were first described in terms of demographic and clinical covariates, followed by a descriptive analysis of data availability on the exposures of interest.

General Linear Model (GLM) analyses were used for each database and for each of the four parameters of interest, to model case-control differences in mean exposure levels separately for each comparison time period and to adjust for covariates. Importantly, each time period comparison was treated as a separate analysis for case-control sets with data present in that time period and covariate adjustments for each comparison, rather than in terms of repeated observations within individuals. The exposures here were modelled as dependent variables, with main independent variables being case-control status, time category, interaction between case-control status and time category, age at index date, gender, the four vascular disorders described above, and stratum variable. Based on plots of unadjusted mean differences by time period, linear time terms were used for DBP as a dependent variable while quadratic time terms were added to models of BMI, SBP and total cholesterol.

Having finalised models for each of the four parameters (BMI, SBP, DBP, total cholesterol) in each EHR dataset, the GLM coefficients for each dataset were pooled in a meta-analysis for each parameter. Random effects were assumed when coefficients were pooled. In secondary analyses, adjusted meta-analysed coefficients for SBP, DBP and total cholesterol were stratified as appropriate according to previously received antihypertensive or lipid lowering medication in cases and controls.



For peer review only - http://bmjopen.bmj.com/site/about/guidelines.xhtml

# RESULTS

Overall, there were 291,780 dementia cases (ranging from 3,433 cases in IMIM-UPF to 139,083 cases in THIN) and 29,170,549 controls made available for analysis from all six EHR databases. As described in Table 1, age and gender were effectively matched in cases and controls. Higher proportions of cases had a history of diabetes, acute myocardial infarction and stroke. Slightly higher proportions of cases had been prescribed antihypertensive, as well as lipid lowering, medication than controls; however, prevalence of recorded hypertension did not differ consistently between cases than controls across the databases. Descriptive data on mean vascular risk factor levels by case/control status, time period and database are provided in Supplementary Table 1, missing data percentages by year and case status in Supplementary Table 2, and missing data by covariates in Supplementary Table 3. Missing data were more prominent for less recent time periods; for time periods over 2 years before the index date, missing data were more common in cases than controls, but more common in controls than cases for the 0-2 year time period; missing data were least common for SBP/DBP and most common for BMI. Mean numbers of missing data points were not found to vary consistently by age or gender, although missing data were consistently below mean levels in those with hypertension and above mean levels in those with stroke.

Figure 1 displays case-control differences in vascular risk factor exposures of interest by time and database. For all exposures apart from DBP, cases had lower values compared with controls (negative case coefficients), all four exposures decreased in later time periods (negative time coefficients) and this was more marked for cases compared to controls (negative case-x-time interaction terms) (Table 2). Quadratic terms were also significant and negative in value for BMI and SBP, reflecting the curvilinear relationships displayed in



For peer review only - http://bmjopen.bmj.com/site/about/guidelines.xhtml

Figure 1, while those for total cholesterol comprised a positive time-squared term and a negative case-x-time-squared interaction, again reflecting the curvilinear visualised relationship. Following adjustment for covariates, case-x-time and case-x-time-squared interaction terms were reduced modestly for BMI and SBP but retained statistical significance. No significant case or case-x-time terms were observed for DBP. For total cholesterol a negative-value case-x-time interaction term persisted, but there was no longer a significant case-x-time-squared interaction.

Following additional stratification of these adjusted models by medication use (Table 3), SBP coefficients were not markedly different (overlapping confidence intervals), although those for case status and the case-x-time interaction were stronger in the subgroups who were not receiving antihypertensive medication; the (negative value) case coefficient for DBP was also stronger in that subgroup. Likewise, case and case-x-time coefficients for total cholesterol levels were stronger in those not receiving lipid lowering medication, although confidence intervals overlapped.

For peer review only



For peer review only - http://bmjopen.bmj.com/site/about/guidelines.xhtml

## DISCUSSION

In this large study spanning 12 years, we compared people with/without a dementia diagnosis and investigated previously recorded levels of BMI, SBP, DBP, and total serum cholesterol. In summary, BMI, SBP and total cholesterol levels were lower overall in cases with dementia than controls and the difference was most marked closest to the point of diagnosis. This time-dependency of the associations was not accounted for by age, gender or recorded vascular disorders, and case-control differences in SBP and total cholesterol did not appear to be modified by antihypertensive or lipid lowering treatment respectively. There was little evidence for associations with DBP after adjustment, and between-database heterogeneity appeared high for associations with total cholesterol, unlike the relative consistency observed with BMI and SBP. As described, while dementia is generally found to be associated with higher levels of vascular risk factors measured 10-20 years before its clinical onset in prospective epidemiological studies, associations are often found to be reversed when investigated closer to time of this clinical onset [16]. The extent to which these associations can be observed in more healthcare data remains to be established, despite opportunities for assessing this over large, 'real world' populations.

Considering BMI, prospective studies with decades follow-up [17] have suggested that midlife obesity is a risk factor for dementia [18], but this association is attenuated or reversed in older age [19], and exaggerated weight loss precedes the clinical onset of dementia [20], described for periods of 2 to 4 years [21], 5 years [22], or up to 10 years [23] before dementia onset. Our findings of lower SBP in cases at the time of diagnosis, and a preceding widening difference over time, are also consistent with epidemiological research, as is the lack of association with DBP: high mid-life SBP is a risk factor for dementia incidence 10-20 years



For peer review only - http://bmjopen.bmj.com/site/about/guidelines.xhtml

For peer review only

later [5, 2, 3], while SBP at dementia clinical onset tends to be lower than controls [5, 24], and exaggerated decline in SBP prior to dementia has been described in Swedish [4, 25] and Japanese American [6] cohorts, as well as during the course of dementia [26]. Although case and case-x-time coefficients were statistically significant for total cholesterol levels, similar to those for BMI and SBP, visual inspection (Figure 1) indicated considerably more inconsistency between databases and we feel that conclusions are less strong in this respect. The relationship between cholesterol level and dementia has been less clear in epidemiological research [27], although tends to support associations with high total cholesterol mid-life but not in late-life. Declining cholesterol levels precede dementia [12, 13], although without the later acceleration described for BMI and SBP, and not observed in all studies [28].

Assuming that the widening gaps in exposure levels between cases and controls in our data reflect dementia-associated decline in these factors, underlying causal pathways may vary. Weight loss might be secondary to early neurodegeneration, reflecting a combination of pre-dementia apathy, loss of initiative, and reduced olfactory function [23]. Cholesterol decline in one study was observed to occur over 10 years before dementia clinical onset, followed by a parallel trajectory [13], so could conceivably be a marker for another risk factor, such as an episode of infection or inflammation resulting in both the decline in cholesterol and increased later risk of dementia. While declining blood pressure might be secondary to early neurodegeneration, it might also represent a risk factor in itself, through reduced cerebral perfusion and watershed ischaemia/infarction, and/or might be a consequence of previous untreated hypertension. The role of antihypertensive treatment in accounting for or modifying pre-dementia blood pressure decline has been controversial. In one cohort, the exaggerated dementia-associated decline in SBP was found to be restricted to people who had not received antihypertensive agents [6], and associations between dementia and lower



For peer review only - http://bmjopen.bmj.com/site/about/guidelines.xhtml

contemporaneous blood pressure were found to be weak or absent in populations from low- and middle-income countries where hypertension is uncommon [29]. However, other cohorts have found stronger contemporaneous associations between dementia and lower blood pressure in people receiving antihypertensives [30] or no modification of later SBP decline [25]. In our databases, the coefficients of interest (i.e. for case and case-x-time) were stronger in the subgroup who had not received antihypertensive treatment, but differences were not substantial and confidence intervals overlapped. The same was true for associations with cholesterol levels, which were also marginally stronger in the subgroup who had not received lipid lowering treatment, although no attempt was made to factor in treatment differences in lipid-lowering effectiveness.

Our study has several strengths. We believe that the data resource used for this analysis is the largest to date by a considerable margin, illustrating the potential value of routine healthcare data and increasing accessibility of electronic health records for research use within robust governance frameworks such as that developed by the EMIF-IMI consortium. In addition, there are advantages in the generalisability of routine data and in patient populations who are less subject to participation selection than research cohorts. Finally, the use of multiple data resources allowed the consistency of associations to be evaluated. This is particularly important for clinical ‘big data’ where large sample sizes result in even very minor associations being identified as statistically significant, and where traditional heterogeneity statistics from meta-analyses are similarly uninformative; this is exemplified in the visualisation of associations with DBP and total cholesterol (Figure 1) where reported analyses from single databases might have given rise to conclusions which are not consistent across others.

The limitations of the study are those that typify routine administrative rather than research data. Measurements were limited to those recorded in routine practice which could be



For peer review only - http://bmjopen.bmj.com/site/about/guidelines.xhtml

harmonised across the six databases, bearing in mind the different clinical coding systems used. For this reason, and as previously described [14] dementia was defined as a composite outcome with no attempt to identify subtypes which are less consistently ascertained in routine clinical practice. Covariates were limited to demographic factors, common disorders, and specific medication groups of interest, and did not include measures of socioeconomic status or lifestyle factors such as smoking status, diet, or physical activity. Missing data are also an important consideration. While the large samples will have reduced the impact of missing data on statistical power, potential bias arising from non-random missingness cannot be excluded, although we feel it is unlikely to account for the findings of interest. As displayed in Supplementary Table 2, the strongest influence on data loss was time, with more missing data in earlier time periods, as would be expected in clinical samples (where routine exposure checks will be more common in older age groups). Therefore, case-control differences at earliest time points should be viewed with caution. However, it is hard to envisage how differential missing data could account for lower exposure levels in future cases, or for the widening of case-control differences over time (since this was not observed for all exposures of interest). Furthermore, influences of age, gender and comorbidity on missingness were relatively minor, apart from understandable higher recording in monitored conditions such as hypertension. Finally, although missing data were more common in cases than controls for most time periods, these differences were not substantial. Consideration was given to missing data when analyses were planned; however, the limited level of covariate ascertainment and potential non-random missingness were felt to preclude imputation approaches.

An important consideration with dementia diagnoses in routine healthcare data from non-specialist services is that there is minimal or no information on the stage/severity of dementia at diagnosis, because of the absence of structured data on cognitive function. While some



For peer review only - http://bmjopen.bmj.com/site/about/guidelines.xhtml

people will have received a dementia diagnosis early in the course of the condition, others may receive this after several years of significant symptoms. Therefore, comparisons are limited with epidemiological cohorts using dementia screening protocols. At least some of the widening difference in exposure levels between cases and controls may therefore have occurred during times when dementia was evident but undiagnosed. Finally, our analyses did not analyse exposure trajectories within individuals, which is the standard approach in epidemiological cohorts. Instead we investigated case-control differences in previously recorded exposures at different times, hypothesising that the dementia-associated trajectories previously reported for these factors would manifest in variation of case-control differences across time periods, and hence case-x-time interaction in the statistical models.

In summary, our observations at group level, there are clear potential implications for clinical services which require further evaluation. For example, there might be good reason for individual trajectories to be more effectively monitored (for example, BMI or SBP change), possibly enhanced by EHR systems that could highlight changes over time and the need for more frequent assessment or supplementary cognitive assessment for earlier risk-identification. In addition, regardless of causation, the potential consequences of low BMI/SBP as comorbidities in dementia have yet to be fully clarified (e.g. as risk factors for falls or functional decline) which again might have implications for future routine monitoring.

For peer review only



For peer review only - http://bmjopen.bmj.com/site/about/guidelines.xhtml

**Figure 1: Mean differences between cases and controls in vascular risk factor exposures of interest by database and time prior to index date**

For peer review only



For peer review only - http://bmjopen.bmj.com/site/about/guidelines.xhtml

**Table 1: Characteristics of component databases: demographic factors and comorbidities**

| | AUH | | HSD | | IPCI | | SIDIAP | | THIN | | IMIM-UPF | |
|---|---|---|---|---|---|---|---|---|---|---|---|---|
| | Case | Control | Case | Control | Case | Control | Case | Control | Case | Control | Case | Control |
| Number | 4,756 | 475,319 | 21,465 | 2,145,779 | 14,039 | 1,401,114 | 109,004 | 10,900,377 | 139,083 | 13,907,094 | 3,433 | 340,866 |
| Mean (SD) age | 78.8 (9.7) | 78.7 (9.7) | 80.6 (8.3) | 80.4 (8.2) | 81.1 (8.7) | 80.9 (8.6) | 81.4 (8.1) | 81.3 (8.1) | 80.9 (8.9) | 80.6 (8.9) | 80.0 (9.6) | 79.8 (9.5) |
| Male gender (%) | 43.0 | 43.0 | 32.8 | 32.8 | 36.7 | 36.7 | 33.8 | 33.8 | 34.8 | 34.8 | 42.1 | 42.0 |
| **Medication use (%)** | | | | | | | | | | | | |
| Antihypertensive | 79.1 | 78.2 | 76.1 | 72.9 | 75.0 | 75.4 | 77.2 | 73.8 | 56.6 | 63.8 | n/a | n/a |
| Lipid lowering | 47.8 | 45.0 | 26.9 | 26.1 | 42.3 | 39.4 | 46.6 | 42.2 | n/a | n/a | n/a | n/a |
| **Comorbidities (%)** | | | | | | | | | | | | |
| Diabetes | 13.0 | 9.5 | 23.1 | 19.8 | 21.2 | 18.9 | 26.6 | 18.9 | 13.0 | 10.7 | 29.7 | 14.0 |
| Hypertension | 40.3 | 33.6 | 63.9 | 61.0 | 48.6 | 52.2 | 64.5 | 64.2 | 49.6 | 52.5 | 61.0 | 37.9 |
| Acute MI | 6.7 | 7.3 | 2.4 | 2.1 | 7.5 | 6.4 | 3.4 | 2.6 | 8.2 | 5.9 | 4.2 | 2.7 |
| Stroke | 13.8 | 7.8 | 8.3 | 6.5 | 12.4 | 7.7 | 12.3 | 6.9 | 10.6 | 5.7 | 12.8 | 3.9 |

n/a: Data not available



For peer review only - http://bmjopen.bmj.com/site/about/guidelines.xhtml

For peer review only

**Table 2: Meta-analysed unadjusted GLM coefficients (with 95% confidence intervals) across component databases for the relationships between case/control status, time period and the four outcomes of interest**

| | BMI | SBP* | DBP*$ | Cholesterol |
|---|---|---|---|---|
| **Unadjusted** | | | | |
| Intercept | 31.6 (31.6, 31.6) | 114.6 (114.5, 114.6) | 84.4 (84.4, 84.4) | 5.4(5.3, 5.5) |
| Case | -1.32 (-1.41, -1.21) | -3.25 (-3.34, -3.16) | 0.04 (-0.03, 0.11) | -0.12 (-0.20, -0.04) |
| Time | -0.11 (-0.17, -0.06) | -1.06 (-1.17, -0.98) | -0.54 (-0.61, -0.45) | -0.03 (-0.05, -0.01) |
| case-x-time | -0.27 (-0.32, -0.19) | -0.63 (-0.72, -0.57) | -0.03 (-0.06, -0.01) | -0.03 (-0.04, -0.02) |
| Time squared | -0.06 (-0.10, -0.02) | -0.13 (-0.19, -0.07) | | 0.01 (0.00, 0.02) |
| case-x-time squared | -0.02 (-0.03, -0.01) | -0.14 (-0.21, -0.10) | | -0.01 (-0.01, -0.00) |
| **Adjusted**** | | | | |
| Intercept | 31.5 (30.9, 32.2) | 117.6 (108.8, 126.5) | 87.0 (81.7, 92.3) | 5.18 (4.89, 5.50) |
| Case | -1.16 (-1.38, -0.93) | -2.83 (-4.49, -1.16) | -0.19 (-0.46, 0.08) | -0.10 (-0.17, -0.04) |
| Time | -0.13 (-0.16, -0.10) | -0.83 (-1.16, -0.50) | -0.50 (-0.57, -0.42) | -0.05 (-0.07, -0.02) |
| case-x-time | -0.18 (-0.28, -0.08) | -0.53 (-0.80, -0.26) | 0.00 (-0.06, 0.06) | -0.05 (-0.09, -0.01) |
| Time squared | -0.01 (-0.08, 0.06) | -0.01 (-0.02, 0.02) | | 0.00 (-0.02, 0.02) |
| case-x-time squared | -0.01 (-0.02, -0.00) | -0.04 (-0.05, -0.02) | | -0.01 (-0.01, 0.01) |

$ Based on linear equation; *SBP and DBP not available in AUH or IMIM-UPF; **GLM coefficients adjusted for age, gender, diabetes, hypertension, acute myocardial infarction, and stroke

For peer review only



For peer review only - http://bmjopen.bmj.com/site/about/guidelines.xhtml

**Table 3: Meta-analysed adjusted GLM coefficients stratified by antihypertensive treatment and lipid lowering medication**

| SBP | With antihypertensive treatment | No antihypertensive treatment |
|---|---|---|
| Intercept | 121.6 (115.5, 127.8) | 109.4 (101.4, 117.5) |
| Case | -2.44 (-3.56, -1.33) | -4.02 (-8.67, 0.63) |
| Time | -1.01 (-1.40, -0.62) | -0.14 (-0.55, 0.28) |
| case-x-time | -0.49 (-0.60, -0.32) | -0.72 (-1.54, 0.10) |
| Time squared | -0.03 (-0.04, -0.01) | -0.04 (-0.05, -0.02) |
| case-x-time squared | -0.04 (-0.05, -0.03) | -0.04 (-0.08, 0.01) |

| DBP | With antihypertensive treatment | No antihypertensive treatment |
|---|---|---|
| Intercept | 89.3 (85.8, 92.9) | 82.3 (76.1, 88.5) |
| Case | -0.05 (-0.43, 0.33) | -1.07 (-1.57, -0.56) |
| Time | -0.55 (-0.64, -0.50) | -0.15 (-0.22, -0.08) |
| case-x-time | 0.02 (-0.02, 0.07) | -0.11 (-0.29, 0.07) |

| Cholesterol | With lipid lowering drugs | No lipid lowering drugs |
|---|---|---|
| Intercept | 5.23 (5.20, 5.25) | 5.44 (5.18, 5.71) |
| Case | 0.02 (-0.01, 0.05) | -0.08 (-0.12, -0.03) |
| Time | -0.07 (-0.10, -0.04) | -0.02 (-0.06, 0.02) |
| case-x-time | -0.01 (-0.01, 0.01) | -0.03 (-0.04, -0.02) |
| Time squared | -0.03 (-0.04, -0.01) | -0.04 (-0.05, -0.02) |
| case-x-time squared | -0.01 (-0.02, 0.00) | -0.01 (-0.01, -0.00) |



For peer review only - http://bmjopen.bmj.com/site/about/guidelines.xhtml

## References


[1] Qiu C., Von Strauss E., Fastbom J., Winblad B., Fratiglioni L. Low blood pressure and risk of dementia in the Kungsholmen project: a 6-year follow-up study. Arch Neurol. 2003; 60: 223–228.

[2] Launer L.J., Ross G.W., Petrovitch H., Masaki K., Foley D., White L.R. et al. Midlife blood pressure and dementia: The Honolulu-Asia Aging study. Neurobiol Aging. 2000;21(1): 49–55.

[3] Kivipelto M., Helkala E.L., Laakso M.P., Hanninen T., Hallikainen M., Alhainen K. et al. (2001) Midlife vascular risk factors and Alzheimer's disease in later life: longitudinal, population based study. BMJ 322: 1447–1451.

[4] Skoog I, Lernfelt B, Landahl S, Palmertz B, Andreasson LA, Nilsson L, Persson G, Oden A, Svanborg A. 15-Year longitudinal study of blood pressure and dementia. Lancet. 1996; 347:1141–1145.

[5] Qiu C, von Strauss E, Winblad B, Fratiglioni L. (2004). Decline in blood pressure over time and risk of dementia: a longitudinal study from the Kungsholmen project. Stroke. 35(8):1810-5.

[6] Stewart R, Xue QL, Masaki K, Petrovitch H, Ross GW, White LR, Launer LJ. Change in blood pressure and incident dementia: a 32-year prospective study. Hypertension. 2009; 54:233–240.

[7] Gustafson DR, Bäckman K, Joas E, Waern M, Östling S, Guo X, Skoog I. 37 years of body mass index and dementia: observations from the Prospective Population Study of Women in Gothenburg, Sweden. J Alzheimer Dis. 2012;28:163–171.

[8] Whitmer R.A., Gustafson D.R., Barrett-Connor E., Haan M.N., Gunderson E.P., Yaffe K. Central obesity and increased risk of dementia more than three decades later. Neurology. 2008;71:1057–1064.

[9] Nourhashemi F, Deschamps V, Larrieu S, et al. Body mass index and incidence of dementia: the PAQUID study. Neurology . 2003.;60(1):117-9.




For peer review only - http://bmjopen.bmj.com/site/about/guidelines.xhtml

For peer review only

[10] Johnson DK, Wilkins CH, Morris JC. Accelerated Weight Loss May Precede Diagnosis in Alzheimer Disease. Arch Neurol. 2006;63(9):1312–1317.

[11] Notkola IL, Sulkava R, Pekkanen J, et al. Serum total cholesterol, apolipoprotein E epsilon 4 allele, and Alzheimer's disease. Neuroepidemiology. 1998;17:14–20.

[12] Mielke, M. M., Zandi, P. P., Shao, H., Waern, M., Östling, S., Guo, X., Björkelund, C., Lissner, L., Skoog, I., … Gustafson, D. R. The 32-year relationship between cholesterol and dementia from midlife to late life. Neurology. 2010;75(21): 1888-95.

[13] Stewart R, White LR, Xue QL, Launer LJ. Twenty-six-year change in total cholesterol levels and incident dementia: the Honolulu-Asia Aging Study. Arch Neurol 2007;64:103–107.

[14] Perera G., Pedersen L., Ansel D., Alexander M., Arrighi H.M., Avillach P., Foskett N., Gini R., Gordon M.F., Gungabissoon U., Mayer M.A., Novak G., Rijnbeek P., Trifirò G., van der Lei J., Visser P.J., Stewart R.(2018) Dementia prevalence and incidence in a federation of European Electronic Health Record databases: The European Medical Informatics Framework resource. Alzheimers Dement. 2018 Feb;14(2):130-139.

[15] Roberto G, Leal I, Sattar N, Loomis AK, Avillach P, Egger P, van Wijngaarden R, Ansell D, Reisberg S, Tammesoo ML, Alavere H, Pasqua A, Pedersen L, Cunningham J, Tramontan L, Mayer MA, Herings R, Coloma P, Lapi F, Sturkenboom M, van der Lei J, Schuemie MJ, Rijnbeek P, Gini R. (2016). Identifying Cases of Type 2 Diabetes in Heterogeneous Data Sources: Strategy from the EMIF Project. 11(8):e0160648

[16] Verghese J, Lipton RB, Hall CB, et al. Low blood pressure and the risk of dementia in very old individuals. Neurology. 2003;61:1667–72.



For peer review only - http://bmjopen.bmj.com/site/about/guidelines.xhtml

[17] Singh-Manoux, A., Fayosse, A., Sabia, S., Tabak, A., Shipley, M., Dugravot, A., & Kivimäki, M. Clinical, socioeconomic, and behavioural factors at age 50 years and risk of cardiometabolic multimorbidity and mortality: A cohort study. PLoS Med. 2018; 15(5): e1002571.

[18] Chuang Y.F., An Y., Bilgel M., Wong D.F., Troncoso J.C., O'Brien R.J. Midlife adiposity predicts earlier onset of Alzheimer's dementia, neuropathology and presymptomatic cerebral amyloid accumulation. Mol Psychiatry. 2016;21:910–915.

[19] Luchsinger J.A., Patel B., Tang M.X., Schupf N., Mayeux R. Measures of adiposity and dementia risk in elderly persons. Arch Neurol. 2007;64:392–398.

[20] Hughes T.F., Borenstein A.R., Schofield E., Wu Y., Larson E.B. Association between late-life body mass index and dementia: the Kame Project. Neurology. 2009;72:1741–1746.

[21] Stewart R., Masaki K., Xue Q.L., Peila R., Petrovitch H., White L.R. A 32-year prospective study of change in body weight and incident dementia: the Honolulu-Asia Aging Study. Arch Neurol. 2005;62:55–60.

[22] Buchman A.S., Wilson R.S., Bienias J.L., Shah R.C., Evans D.A., Bennett D.A. Change in body mass index and risk of incident Alzheimer disease. Neurology. 2005; 65:892–897.

[23] Knopman D.S., Edland S.D., Cha R.H., Petersen R.C., Rocca W.A. Incident dementia in women is preceded by weight loss by at least a decade. Neurology. 2007;69:739–746.

[24] Guo X, Pantoni L, Simoni M, Bengtsson C, Bjorkelund C, Lissner L, Gustafson D, Skoog I. Blood pressure components and changes in relation to white matter lesions: a 32-year prospective population study. Hypertension. 2009;54:57–62.

[25] Joas E., Bäckman K., Gustafson D., Ostling S., Waern M., Guo X., Skoog I. Blood pressure trajectories from midlife to late life in relation to dementia in women followed for 37 years. Hypertension. 2012;59:796-801.

For peer review only



For peer review only - http://bmjopen.bmj.com/site/about/guidelines.xhtml

[26] Hanon O, Latour F, Seux M, Lenoir H, Forette F, Rigaud A, Real F. Evolution of blood pressure in patients with Alzheimer's disease: a one year survey of a French cohort. J Nutr Health Aging. 2005;9:106–111.

[27] Anstey, K. J., Ashby-Mitchell, K., & Peters, R. Updating the Evidence on the Association between Serum Cholesterol and Risk of Late-Life Dementia: Review and Meta-Analysis. Journal of Alzheimer's disease : JAD. 2017; 56(1): 215-228.

[28] Wagner M, Helmer C, Tzourio C, Berr C, Proust-Lima C, Samieri C. Evaluation of the Concurrent Trajectories of Cardiometabolic Risk Factors in the 14 Years Before Dementia. JAMA Psychiatry. 2018;75(10):1033–1042.

[29] Albanese, E., Lombardo, F. L., Prince, M. J., & Stewart, R. Dementia and lower blood pressure in Latin America, India, and China: a 10/66 cross-cohort study. Neurology. 2013; 81(3): 228-35.

[30] Ruitenberg A, Skoog I, Ott A, Aevarsson O, Witteman J, C, M, Lernfelt B, van Harskamp F, Hofman A, Breteler M, M, B: Blood Pressure and Risk of Dementia: Results from the Rotterdam Study and the Gothenburg H-70 Study. Dement Geriatr Cogn Disord. 2001;12:33-3

For peer review only

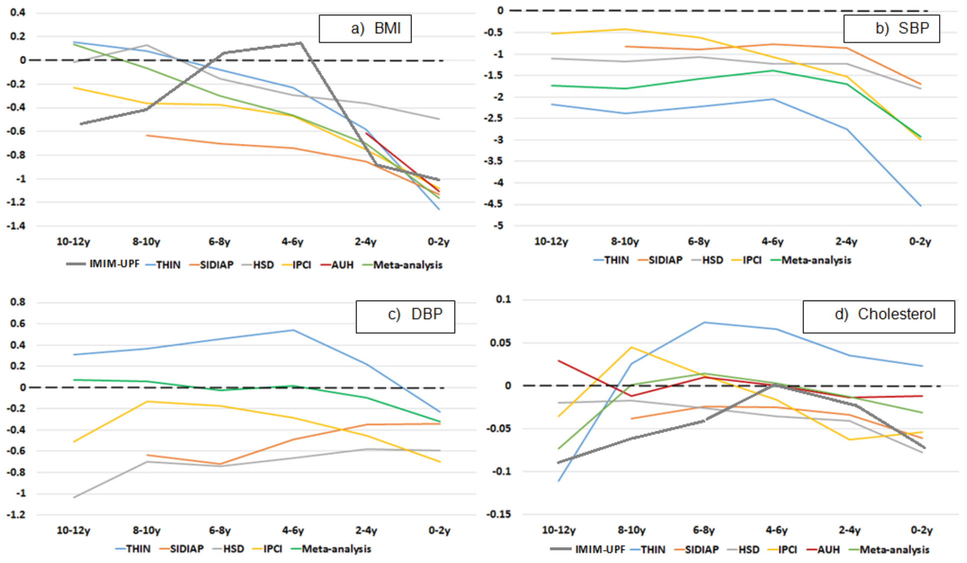


Figure 1: Mean differences between cases and controls in vascular risk factor exposures of interest by database and time prior to index date

**Supplementary Table 1: Vascular risk factor status at each measurement period by database**

| Risk factors | Case/ Control status | AUH | | | | | |
|---|---|---|---|---|---|---|---|
| | | 0- 2 Years* | 2- 4 years | 4- 6 years | 6- 8 years | 8- 10 years | 10- 12 years |
| Mean BMI (SD) | Case | 24.27 (4.56) | 24.96 (4.74) | 26.03 (4.36) | | | |
| | Control | 25.38 (4.61) | 25.56 (4.46) | 25.40 (4.49) | | | |
| Mean Total cholesterol (SD) | Case | 4.86 (1.10) | 4.99 (1.09) | 5.11 (1.06) | 5.27 (1.07) | 5.44 (1.03) | 5.75 (1.10) |
| | Control | 4.87 (1.06) | 5.00 (1.05) | 5.12 (1.05) | 5.26 (1.08) | 5.46 (1.07) | 5.72 (1.04) |

| Risk factors | Case/ Control status | HSD | | | | | |
|---|---|---|---|---|---|---|---|
| | | 0- 2 Years | 2- 4 years | 4- 6 years | 6- 8 years | 8- 10 years | 10- 12 years |
| Mean BMI (SD) | Case | 26.40 (4.89) | 26.71 (4.87) | 26.98 (4.79) | 27.29 (4.72) | 27.59 (4.76) | 27.33 (4.59) |
| | Control | 26.90 (4.38) | 27.07 (4.25) | 27.27 (4.21) | 27.44 (4.25) | 27.46 (4.25) | 27.35 (4.39) |
| Mean SBP (SD) | Case | 137.22 (15.64) | 139.44 (15.38) | 140.72 (15.39) | 141.99 (15.28) | 142.95 (15.47) | 143.84 (15.78) |
| | Control | 139.03 (14.69) | 140.65 (14.93) | 141.95 (15.12) | 143.07 (15.26) | 144.11 (15.23) | 144.95 (15.49) |

For peer review only

| | | | | | | | |
|---|---|---|---|---|---|---|---|
| Mean DBP (SD) | Case | 78.83 (7.38) | 79.72 (7.31) | 80.55 (7.36) | 81.34 (7.40) | 82.17 (7.42) | 82.97 (7.70) |
| | Control | 78.24 (7.71) | 79.16 (7.45) | 79.89 (7.29) | 80.59 (7.30) | 81.48 (7.42) | 81.93 (7.56) |
| Mean Total cholesterol (SD) | Case | 5.39 (1.04) | 5.51 (1.02) | 5.63 (1.01) | 5.73 (1.01) | 5.85 (0.99) | 5.95 (0.98) |
| | Control | 5.31 (1.10) | 5.47 (1.08) | 5.59 (1.06) | 5.70 (1.05) | 5.83 (1.03) | 5.93 (1.02) |

| Risk factors | Case/ Control status | IPCI | | | | | |
|---|---|---|---|---|---|---|---|
| | | 0- 2 Years | 2- 4 years | 4- 6 years | 6- 8 years | 8- 10 years | 10- 12 years |
| Mean BMI (SD) | Case | 26.25 (4.54) | 26.80 (4.41) | 27.11 (4.37) | 27.26 (4.45) | 27.48 (4.62) | 27.78 (4.64) |
| | Control | 27.32 (4.54) | 27.54 (4.61) | 27.57 (4.63) | 27.62 (4.74) | 27.83 (4.74) | 28.01 (4.64) |
| Mean SBP (SD) | Case | 141.25 (17.75) | 144.34 (17.39) | 146.22 (17.24) | 147.98 (17.74) | 149.65 (17.80) | 150.35 (17.94) |
| | Control | 144.28 (16.89) | 145.88 (16.90) | 147.31 (16.94) | 148.63 (17.03) | 150.08 (17.25) | 150.86 (17.86) |
| Mean DBP (SD) | Case | 77.75 (8.81) | 79.02 (8.61) | 80.23 (8.53) | 81.41 (8.26) | 82.75 (8.39) | 83.68 (8.40) |
| | Control | 78.45 (8.48) | 79.47 (8.48) | 80.52 (8.41) | 81.59 (8.40) | 82.88 (8.28) | 84.18 (8.16) |
| Mean Total cholesterol (SD) | Case | 5.01 (1.15) | 5.12 (1.15) | 5.23 (1.16) | 5.34 (1.17) | 5.52 (1.11) | 5.70 (1.11) |
| | Control | 5.07 (1.12) | 5.18 (1.14) | 5.24 (1.19) | 5.33 (1.17) | 5.48 (1.13) | 5.73 (1.01) |

For peer review only

| Risk factors | Case/ Control status | SIDIAP | | | | | |
|---|---|---|---|---|---|---|---|
| | | 0- 2 Years | 2- 4 years | 4- 6 years | 6- 8 years | 8- 10 years | 10- 12 years |
| Mean BMI (SD) | Case | 27.54 (4.55) | 28.11 (4.49) | 28.49 (4.45) | 28.79 (4.47) | 29.10 (4.39) | |
| | Control | 28.67 (4.68) | 28.97 (4.61) | 29.23 (4.56) | 29.49 (4.54) | 29.74 (4.53) | |
| Mean SBP (SD) | Case | 134.19 (14.33) | 136.10 (14.18) | 136.95 (14.36) | 137.33 (14.54) | 137.73 (15.19) | |
| | Control | 135.90 (13.03) | 136.96 (13.43) | 137.73 (13.78) | 138.23 (14.08) | 138.56 (14.24) | |
| Mean DBP (SD) | Case | 73.15 (8.17) | 74.21 (8.00) | 75.02 (7.95) | 75.75 (7.88) | 76.49 (8.18) | |
| | Control | 73.49 (7.99) | 74.56 (7.98) | 75.51 (7.90) | 76.47 (7.91) | 77.12 (7.90) | |
| Mean Total cholesterol (SD) | Case | 5.10 (0.99) | 5.22 (0.96) | 5.30 (0.94) | 5.35 (0.93) | 5.39 (0.93) | |
| | Control | 5.16 (0.98) | 5.26 (0.97) | 5.32 (0.94) | 5.38 (0.93) | 5.43 (0.93) | |

| Risk factors | Case/ Control status | THIN | | | | | |
|---|---|---|---|---|---|---|---|
| | | 0- 2 Years | 2- 4 years | 4- 6 years | 6- 8 years | 8- 10 years | 10- 12 years |
| Mean BMI (SD) | Case | 24.95 (5.19) | 25.75 (5.03) | 26.2 (8.90) | 26.47 (4.87) | 26.66 (4.83) | 26.74 (4.77) |

For peer review only

| | | | | | | | |
|---|---|---|---|---|---|---|---|
| | Control | 26.18 (4.89) | 26.32 (4.75) | 26.41 (4.68) | 26.53 (4.60) | 26.57 (4.54) | 26.57 (4.49) |
| Mean SBP (SD) | Case | 136.75 (17.34) | 140.59 (17.84) | 142.98 (17.96) | 144.23 (18.32) | 145.11 (19.00) | 146.09 (19.46) |
| | Control | 141.29 (16.61) | 143.35 (16.74) | 145.04 (17.04) | 146.46 (17.44) | 147.47 (17.80) | 148.22 (18.33) |
| Mean DBP (SD) | Case | 75.72 (9.13) | 77.21 (9.28) | 78.47 (9.30) | 79.44 (9.37) | 80.36 (8.64) | 81.39 (9.69) |
| | Control | 75.93 (8.42) | 76.99 (8.43) | 77.93 (8.54) | 78.97 (8.69) | 80.00 (8.73) | 81.05 (8.88) |
| Mean Total cholesterol (SD) | Case | 4.75 (1.19) | 4.82 (1.22) | 4.89 (1.25) | 4.98 (1.30) | 5.07 (1.38) | 5.20 (1.52) |
| | Control | 4.73 (1.18) | 4.79 (1.20) | 4.83 (1.24) | 4.90 (1.28) | 5.05 (1.31) | 5.32 (1.31) |

| Risk factors | Case/ Control status | IMIM-UPF | | | | | |
|---|---|---|---|---|---|---|---|
| | | 0- 2 Years | 2- 4 years | 4- 6 years | 6- 8 years | 8- 10 years | 10- 12 years |
| Mean BMI (SD) | Case | 26.88 (4.63) | 27.06 (4.43) | 29.01 (4.92) | 29.33 (5.13) | 28.69 (4.50) | |
| | Control | 27.92 (4.51) | 28.42 (4.58) | 28.85 (4.49) | 29.28 (4.86) | 30.35 (4.70) | |
| Mean Total cholesterol (SD) | Case | 4.27 (1.08) | 4.84 (1.15) | 5.01 (1.17) | 5.14 (1.15) | 5.26 (1.09) | 5.31 (1.09) |
| | Control | 4.71 (1.12) | 4.86 (1.13) | 5.01 (1.12) | 5.18 (1.12) | 5.35 (1.10) | 5.50 (1.07) |

*Time periods (years prior to index date)

For peer review only

**Supplementary Table 2: Proportions of patients with missing data for each vascular risk factor in each database**

| All databases | | 0-2* | 2-4 | 4-6 | 6-8 | 8-10 | 10- 12 |
|---|---|---|---|---|---|---|---|
| BMI | Cases | 52% | 61% | 69% | 77% | 85% | 91% |
| | Control | 56% | 60% | 67% | 75% | 81% | 87% |
| SBP | Cases | 19% | 34% | 47% | 59% | 70% | 78% |
| | Control | 22% | 29% | 40% | 51% | 62% | 70% |
| DBP | Cases | 19% | 34% | 47% | 59% | 70% | 78% |
| | Control | 22% | 29% | 40% | 51% | 62% | 70% |
| Tchol | Cases | 39% | 53% | 64% | 75% | 85% | 92% |
| | Control | 43% | 51% | 61% | 72% | 82% | 89% |

| AUH | | 0-2 | 2-4 | 4-6 | 6-8 | 8-10 | 10- 12 |
|---|---|---|---|---|---|---|---|
| BMI | Cases | 50% | 87% | 99% | | | |
| | Control | 62% | 84% | 98% | | | |
| Tchol | Cases | 23% | 38% | 44% | 55% | 65% | 78% |
| | Control | 29% | 35% | 44% | 55% | 68% | 79% |

| HSD | | 0-2 | 2-4 | 4-6 | 6-8 | 8-10 | 10- 12 |
|---|---|---|---|---|---|---|---|
| BMI | Cases | 78% | 82% | 85% | 88% | 91% | 94% |
| | Control | 79% | 82% | 85% | 88% | 91% | 94% |
| SBP | Cases | 47% | 52% | 57% | 64% | 71% | 79% |
| | Control | 49% | 52% | 57% | 64% | 72% | 80% |
| DBP | Cases | 47% | 52% | 57% | 63% | 71% | 79% |
| | Control | 49% | 52% | 57% | 64% | 72% | 80% |

For peer review only

| | | | | | | | |
|---|---|---|---|---|---|---|---|
| Tchol | Cases | 54% | 60% | 64% | 70% | 77% | 83% |
| | Control | 53% | 57% | 63% | 69% | 76% | 82% |

| IPCI | | 0-2 | 2-4 | 4-6 | 6-8 | 8-10 | 10- 12 |
|---|---|---|---|---|---|---|---|
| BMI | Cases | 67% | 72% | 76% | 83% | 88% | 93% |
| | Control | 65% | 70% | 75% | 82% | 88% | 92% |
| SBP | Cases | 18% | 29% | 36% | 45% | 53% | 59% |
| | Control | 21% | 27% | 33% | 41% | 48% | 54% |
| DBP | Cases | 18% | 29% | 36% | 45% | 53% | 60% |
| | Control | 21% | 27% | 41% | 41% | 48% | 55% |
| Tchol | Cases | 55% | 58% | 63% | 70% | 77% | 83% |
| | Control | 50% | 53% | 48% | 67% | 77% | 83% |

| SIDIAP | | 0-2 | 2-4 | 4-6 | 6-8 | 8-10 | 10- 12 |
|---|---|---|---|---|---|---|---|
| BMI | Cases | 45% | 54% | 67% | 80% | 93% | |
| | Control | 46% | 55% | 68% | 82% | 93% | |
| SBP | Cases | 20% | 35% | 54% | 74% | 90% | |
| | Control | 24% | 36% | 56% | 75% | 90% | |
| DBP | Cases | 20% | 54% | 54% | 74% | 90% | |
| | Control | 24% | 99% | 56% | 75% | 90% | |
| Tchol | Cases | 24% | 41% | 60% | 77% | 92% | |
| | Control | 32% | 44% | 61% | 78% | 93% | |

| THIN | | 0-2 | 2-4 | 4-6 | 6-8 | 8-10 | 10- 12 |
|---|---|---|---|---|---|---|---|
| BMI | Cases | 52% | 60% | 67% | 72% | 77% | 83% |

For peer review only

| | | | | | | | |
|---|---|---|---|---|---|---|---|
| | Control | 57% | 58% | 60% | 65% | 69% | 74% |
| SBP | Cases | 16% | 34% | 44% | 52% | 60% | 68% |
| | Control | 18% | 22% | 28% | 35% | 42% | 50% |
| DBP | Cases | 16% | 34% | 44% | 52% | 60% | 68% |
| | Control | 18% | 22% | 28% | 35% | 42% | 50% |
| Tchol | Cases | 48% | 61% | 69% | 75% | 82% | 88% |
| | Control | 50% | 55% | 61% | 68% | 76% | 83% |

| IMIM-UPF | | 0-2 | 2-4 | 4-6 | 6-8 | 8-10 | 10- 12 |
|---|---|---|---|---|---|---|---|
| BMI | Cases | 94% | 98% | 98% | 99% | 99% | |
| | Control | 95% | 97% | 98% | 99% | 99% | |
| Tchol | Cases | 31% | 64% | 70% | 76% | 81% | 86% |
| | Control | 68% | 71% | 75% | 79% | 83% | 87% |

*Time periods (years prior to index date)

For peer review only

**Supplementary Table 3:** **Meta-analysed mean number of missing data points* (95% confidence intervals)**

| | BMI | | SBP | | DBP | | Total cholesterol | |
|---|---|---|---|---|---|---|---|---|
| **Variable** | **Control** | **Case** | **Control** | **Case** | **Control** | **Case** | **Control** | **Case** |
| **Age at index date** | | | | | | | | |
| Under 60 | 2.75 (1.74, 3.76) | 2.54 (1.13, 3.95) | 2.78 (1.77, 3.79) | 2.77 (1.68, 3.86) | 2.78 (1.77, 3.79) | 2.77 (1.68, 3.86) | 2.67 (1.42, 3.92) | 2.58 (1.46, 3.70) |
| 60- 69 | 2.81 (1.74, 3.88) | 2.76 (1.28, 4.24) | 2.75 (1.77, 3.73) | 2.73 (1.50, 3.96) | 2.75 (1.77, 3.73) | 2.73 (1.50, 3.96) | 2.89 (1.43, 4.35) | 2.29 (0.84, 3.74) |
| 70- 79 | 2.89 (1.83, 3.95) | 2.84 (1.02, 4.66) | 2.81 (1.63, 3.99) | 2.85 (1.56, 4.14) | 2.81 (1.63, 3.99) | 2.85 (1.56, 4.14) | 2.28 (1.27, 3.29) | 2.44 (1.15, 3.73) |
| 80- 89 | 2.81 (1.25, 4.37) | 2.86 (1.3, 4.42) | 2.17 (0.85, 3.49) | 2.67 (1.51, 3.83) | 2.17 (0.85, 3.49) | 2.67 (1.51, 3.83) | 2.76 (1.55, 3.97) | 2.54 (1.22, 3.86) |
| 90 and over | 2.54 (1.13, 3.95) | 2.65 (1.16, 4.14) | 2.71 (1.26, 4.16) | 2.45 (1.16, 3.74) | 2.71 (1.26, 4.16) | 2.45 (1.16, 3.74) | 2.44 (1.10, 3.78) | 2.77 (1.42, 4.12) |
| **Gender** | | | | | | | | |
| Male | 2.78 (1.19, 4.37) | 2.71 (1.08, 4.34) | 2.60 (1.51, 3.69) | 2.74 (1.69, 3.79) | 2.60 (1.51, 3.69) | 2.74 (1.69, 3.79) | 2.24 (1.23, 3.25) | 2.56 (1.47, 3.65) |
| Female | 2.71 (1.28, 4.14) | 2.75 (1.17, 4.33) | 2.69 (1.37, 4.01) | 2.64 (1.38, 3.90) | 2.69 (1.37, 4.01) | 2.64 (1.38, 3.90) | 2.32 (1.09, 3.55) | 2.48 (1.34, 3.62) |
| **Co-morbidities** | | | | | | | | |
| AMI | 2.55 (1.47, 3.63) | 2.63 (1.38, 3.88) | 3.01 (1.94, 4.08) | 2.97 (1.85, 4.09) | 3.01 (1.94, 4.08) | 2.97 (1.85, 4.09) | 2.12 (0.88, 3.36) | 2.10 (0.64, 3.56) |
| DM | 2.76 (1.62, 3.90) | 2.71 (1.35, 4.07) | 2.70 (1.45, 3.95) | 2.75 (1.63, 3.87) | 2.70 (1.45, 3.95) | 2.75 (1.63, 3.87) | 2.07 (0.62, 3.52) | 2.13 (0.77, 3.49) |

For peer review only

| | | | | | | | | |
|---|---|---|---|---|---|---|---|---|
| Stroke | 2.82 (1.59, 4.05) | 2.78 (1.32, 4.24) | 2.81 (1.36, 4.26) | 2.72 (1.49, 3.95) | 2.81 (1.36, 4.26) | 2.72 (1.49, 3.95) | 2.87 (1.01, 4.73) | 2.39 (0.72, 4.06) |
| Hypertension | 2.12 (1.11, 3.13) | 2.05 (0.80, 3.30) | 2.43 (1.32, 3.54) | 2.39 (1.38, 3.40) | 2.43 (1.32, 3.54) | 2.39 (1.38, 3.40) | 2.19 (0.78, 3.60) | 2.14 (0.55, 3.73) |
| Total sample | 2.75 | 2.73 | 2.67 | 2.66 | 2.67 | 2.66 | 2.30 | 2.50 |

*From a denominator of 6 data points apart from the following: UPF-BMI 5 data points; AUH-BMI 3 data points; SIDIAP variables 5 data points

AMI: acute myocardial infarction; DM: type 2 diabetes mellitus

For peer review only

## STROBE 2007 (v4) Statement—Checklist of items that should be included in reports of *case-control studies*

| Section/Topic | Item # | Recommendation | Reported on page # |
|---|---|---|---|
| **Title and abstract** | 1 | (*a*) Indicate the study's design with a commonly used term in the title or the abstract | 5 |
| | | (*b*) Provide in the abstract an informative and balanced summary of what was done and what was found | 5- 6 |
| **Introduction** | | | |
| Background/rationale | 2 | Explain the scientific background and rationale for the investigation being reported | 8 |
| Objectives | 3 | State specific objectives, including any prespecified hypotheses | 8- 9 |
| **Methods** | | | |
| Study design | 4 | Present key elements of study design early in the paper | 10 |
| Setting | 5 | Describe the setting, locations, and relevant dates, including periods of recruitment, exposure, follow-up, and data collection | 10- 12 |
| Participants | 6 | *(*a) Give the eligibility criteria, and the sources and methods of case ascertainment and control selection. Give the rationale for the choice of cases and controls | 10- 11 |
| | | (*b*) For matched studies, give matching criteria and the number of controls per case | 10- 11 |
| Variables | 7 | Clearly define all outcomes, exposures, predictors, potential confounders, and effect modifiers. Give diagnostic criteria, if applicable | 11 |
| Data sources/ measurement | 8* | For each variable of interest, give sources of data and details of methods of assessment (measurement). Describe comparability of assessment methods if there is more than one group | 11 |
| Bias | 9 | Describe any efforts to address potential sources of bias | 18, Supplementary table 2 |
| Study size | 10 | Explain how the study size was arrived at | 10 |
| Quantitative variables | 11 | Explain how quantitative variables were handled in the analyses. If applicable, describe which groupings were chosen and why | 12 |
| Statistical methods | 12 | (*a*) Describe all statistical methods, including those used to control for confounding | |
| | | (*b*) Describe any methods used to examine subgroups and interactions | 12 |
| | | (*c*) Explain how missing data were addressed | 12 and Supplementary table 2 |

For peer review only

| | | | |
|---|---|---|---|
| | | (*d*) If applicable, explain how matching of cases and controls was addressed | 10- 11 |
| | | (*e*) Describe any sensitivity analyses | |
| **Results** | | | |
| Participants | 13* | (a) Report numbers of individuals at each stage of study—eg numbers potentially eligible, examined for eligibility, confirmed eligible, included in the study, completing follow-up, and analysed | 13- 14, supplementary table 2 and 3 |
| | | (b) Give reasons for non-participation at each stage | 13- 14, supplementary table 2 and 3 |
| | | (c) Consider use of a flow diagram | |
| Descriptive data | 14* | (a) Give characteristics of study participants (eg demographic, clinical, social) and information on exposures and potential confounders | Table 1 in page 21 |
| | | (b) Indicate number of participants with missing data for each variable of interest | supplementary table 2 |
| Outcome data | 15* | Report numbers in each exposure category, or summary measures of exposure | Table 1 and table 2 in page 21 and 22 and Supplementary Table 1 |
| Main results | 16 | (*a*) Give unadjusted estimates and, if applicable, confounder-adjusted estimates and their precision (eg, 95% confidence interval). Make clear which confounders were adjusted for and why they were included | Table 2 and table 3 in page 22 and 23 |
| | | (*b*) Report category boundaries when continuous variables were categorized | Page 11 |
| | | (*c*) If relevant, consider translating estimates of relative risk into absolute risk for a meaningful time period | |
| Other analyses | 17 | Report other analyses done—eg analyses of subgroups and interactions, and sensitivity analyses | Table 3 for subgroup analysis in page 23; |
| **Discussion** | | | |
| Key results | 18 | Summarise key results with reference to study objectives | Page 15 |
| Limitations | 19 | Discuss limitations of the study, taking into account sources of potential bias or imprecision. | Page 17-18 |

For peer review only

| | | | |
|---|---|---|---|
| | | Discuss both direction and magnitude of any potential bias | |
| Interpretation | 20 | Give a cautious overall interpretation of results considering objectives, limitations, multiplicity of analyses, results from similar studies, and other relevant evidence | Page 18- 19 |
| Generalisability | 21 | Discuss the generalisability (external validity) of the study results | Page 18- 19 |
| **Other information** | | | |
| Funding | 22 | Give the source of funding and the role of the funders for the present study and, if applicable, for the original study on which the present article is based | Page 2 |

*Give information separately for cases and controls in case-control studies and, if applicable, for exposed and unexposed groups in cohort and cross-sectional studies.

**Note:** An Explanation and Elaboration article discusses each checklist item and gives methodological background and published examples of transparent reporting. The STROBE checklist is best used in conjunction with this article (freely available on the Web sites of PLoS Medicine at http://www.plosmedicine.org/, Annals of Internal Medicine at http://www.annals.org/, and Epidemiology at http://www.epidem.com/). Information on the STROBE Initiative is available at www.strobe-statement.org.

For peer review only

BMJ Open

# Vascular and metabolic risk factor differences prior to dementia diagnosis: a multi-database case control study using European electronic health records



| | |
|---|---|
| Journal: | *BMJ Open* |
| Manuscript ID | bmjopen-2020-038753.R1 |
| Article Type: | Original research |
| Date Submitted by the Author: | 16-Jun-2020 |





| | |
|---|---|
| Complete List of Authors: | Perera, Gayan; Institute of Psychiatry Psychology and Neuroscience, Psychological Medicine<br>Rijnbeek, P; Erasmus Medical Center<br>Alexander, Myriam; GlaxoSmithKline Plc, Quantitative Sciences, , Uxbridge, United Kingdom.<br>Ansell, David; University of Birmingham, Institute of Applied Health Research<br>Avillach, Paul; Harvard Medical School<br>Duarte-Salles, Talita; IDIAP Jordi Gol<br>Gordon, Mark ; Teva Pharmaceuticals USA Inc, Specialty Clinical Development, Neurology and Psychiatry<br>Lapi, Francesco; Italian College of General Practitioners and Primary Care<br>Mayer, Miguel; Hospital del Mar Institute for Medical Research<br>Pasqua, Alessandro; Health Search, Italian College of General Practitioners and Primary Care<br>Pedersen, Lars; Aarhus University Hospital, Department of Clinical Epidemiology<br>van Der Lei, Johan; Erasmus Medical Center<br>Visser, Pieter; Maastricht University, Department of Psychiatry and Neuropsychology<br>Stewart, Robert; Institute of Psychiatry Psychology and Neuroscience, Psychological Medicine |





| | |
|---|---|
| <b>Primary Subject Heading</b>: | Mental health |
| Secondary Subject Heading: | Epidemiology, Geriatric medicine, Health informatics, Public health, Research methods |
| Keywords: | Dementia < NEUROLOGY, Physiology < NATURAL SCIENCE DISCIPLINES, Health informatics < BIOTECHNOLOGY & BIOINFORMATICS, Hypertension < CARDIOLOGY, EPIDEMIOLOGY, MENTAL HEALTH |
| | |

SCHOLARONE™
Manuscripts

*I, the Submitting Author has the right to grant and does grant on behalf of all authors of the Work (as defined in the below author licence), an exclusive licence and/or a non-exclusive licence for contributions from authors who are: i) UK Crown employees; ii) where BMJ has agreed a CC-BY licence shall apply, and/or iii) in accordance with the terms applicable for US Federal Government officers or employees acting as part of their official duties; on a worldwide, perpetual, irrevocable, royalty-free basis to BMJ Publishing Group Ltd ("BMJ") its licensees and where the relevant Journal is co-owned by BMJ to the co-owners of the Journal, to publish the Work in this journal and any other BMJ products and to exploit all rights, as set out in our licence.*

*The Submitting Author accepts and understands that any supply made under these terms is made by BMJ to the Submitting Author unless you are acting as an employee on behalf of your employer or a postgraduate student of an affiliated institution which is paying any applicable article publishing charge ("APC") for Open Access articles. Where the Submitting Author wishes to make the Work available on an Open Access basis (and intends to pay the relevant APC), the terms of reuse of such Open Access shall be governed by a Creative Commons licence – details of these licences and which Creative Commons licence will apply to this Work are set out in our licence referred to above.*

*Other than as permitted in any relevant BMJ Author's Self Archiving Policies, I confirm this Work has not been accepted for publication elsewhere, is not being considered for publication elsewhere and does not duplicate material already published. I confirm all authors consent to publication of this Work and authorise the granting of this licence.*

For peer review only

**Vascular and metabolic risk factor differences prior to dementia diagnosis: a multi-database case control study using European electronic health records**

Gayan Perera[1], PhD; Peter R. Rijnbeek[2], PhD; Myriam Alexander[3], PhD; David Ansell[4], MD PhD; Paul Avillach[5,6], MD, PhD; Talita Duarte-Salles, MPH, PhD[7]; Mark Forrest Gordon[8], MD; Francesco Lapi[9], PhD; Miguel A. Mayer[10], MD, PhD; Alessandro Pasqua[9], MSc; Lars Pedersen[11], PhD; Johan van der Lei[2], PhD, MD; Pieter Jelle Visser[12,13,14], MD PhD; Robert Stewart[1,15], MD, FRCPsych.

[1]King's College London (Institute of Psychiatry, Psychology and Neuroscience), London, United Kingdom.

[2]Department of Medical Informatics, Erasmus Medical Center, Rotterdam, Netherlands

[3]Quantitative Sciences, GlaxoSmithKline, Uxbridge, United Kingdom.

[4]Institute of Applied Health Research, University of Birmingham, Birmingham, UK

[5]Aarhus University, Aarhus, Denmark

[6]Department of Biomedical Informatics, Harvard Medical School, Boston, MA, USA

[7]Fundació Institut Universitari per a la recerca a l'Atenció Primària de Salut Jordi Gol i Gurina (IDIAPJGol), Barcelona, Spain

[8]Specialty Clinical Development, Neurology and Psychiatry, Teva Pharmaceuticals, Frazer, PA 19355 USA

[9]Health Search, Italian College of General Practitioners and Primary Care, Florence, Italy



For peer review only - http://bmjopen.bmj.com/site/about/guidelines.xhtml

[10]Universitat Pompeu Fabra and Hospital del Mar Medical Research Institute, Barcelona, Spain

[11]Department of Clinical Epidemiology, Aarhus University Hospital, Aarhus, Denmark

[12]Alzheimer Center Amsterdam, Department of Neurology, Amsterdam Neuroscience, Amsterdam UMC, Vrije Universiteit, Amsterdam, The Netherlands

[13]Department of Psychiatry and Neuropsychology, School for Mental Health and Neuroscience (MHeNS), Alzheimer Centrum Limburg, Maastricht University, Maastricht, The Netherlands

[14]Department of Neurobiology, Care Sciences and Society, Division of Neurogeriatrics, Karolinska Institutet, Stockholm Sweden.

[15]South London and Maudsley NHS Foundation Trust, London, United Kingdom

**Corresponding author:** Robert Stewart, Department of Psychological Medicine (Box 84), Institute of Psychiatry, Psychology and Neuroscience (King's College London), De Crespigny Park, London SE5 8AF, UK. Phone: +44 (0)20 7848 0778, Email: robert.stewart@kcl.ac.uk

**Funding statement:** The research leading to these results has received support from the Innovative Medicines Initiative (IMI) Joint Undertaking under EMIF grant agreement n° 115372, resources of which are composed of financial contribution from the European Union's Seventh Framework Programme (FP7/2007-2013) and EFPIA companies' in kind contribution. RS and GP are part-funded by the National Institute for Health Research



For peer review only - http://bmjopen.bmj.com/site/about/guidelines.xhtml

(NIHR) Biomedical Research Centre and Dementia Biomedical Research Unit at South London and Maudsley NHS Foundation Trust and King's College London.

**Competing interests statement:**

MA received funding from Roche and GSK as a contracted employee; GP received funding from Roche as contracted employee during this project; MFG has prior employment at Boehringer Ingelheim Pharmaceuticals, and is a current employee of Teva Pharmaceuticals; RS has received research funding from Roche, Janssen, GSK and Takeda.

**Authors' contributions:**

The analysis was conceived and designed by RS, PRR, PJV, MA, MFG, FL and JVDL. Data extractions and advice on analysis design were provided by the data controllers (DA, TD-S, MAM, AP, LP, JVDL) in liaison with PRR and PA. Analyses were carried out by LP and GP. The manuscript was written by GP and RS. Further comments and significant input were obtained from all co-authors who also oversaw the planning and development of the project.

**Acknowledgements**

(see under funding, above)

**Data sharing statement:** No additional data are available. The datasets analysed during the current study are not publicly available due to confidentially of patients but are available via application to individual data custodians under the EMIF platform (http://www.emif.eu/). The data provided by each database owners were already anonymised.



For peer review only - http://bmjopen.bmj.com/site/about/guidelines.xhtml

For peer review only

**Licence statement:** This is an open access article distributed in accordance with the Creative Commons Attribution Non Commercial (CC BY-NC 4.0) license, which permits others to distribute, remix, adapt, build upon this work non-commercially, and license their derivative works on different terms, provided the original work is properly cited, appropriate credit is given, any changes made indicated, and the use is non-commercial. See: http://creativecommons.org/licenses/by-nc/4.0/.

**Manuscript word count:** 3020

For peer review only



For peer review only - http://bmjopen.bmj.com/site/about/guidelines.xhtml

## ABSTRACT

OBJECTIVE: The objective of the study is to compare body mass index (BMI), systolic/diastolic blood pressure (SBP/DBP), and serum total cholesterol levels between dementia cases and controls at multiple time intervals prior to dementia onset, and to test time interval as a modifying factor for these associations.

DESIGN: Case control study.

SETTING: Six European electronic health records databases.

PARTICIPANTS: 291,780 cases at the date of first-recorded dementia diagnosis, compared to 29,170,549 controls randomly selected from the same databases, age- and sex-matched at this index date.

EXPOSURE: The following measures were extracted whenever recorded within each dataset: BMI (kg/m$^2$), SBP and DBP (mmHg), and serum total cholesterol (mmol/l). Levels for each of these variables were defined within six 2-year time intervals over the 12 years prior to the index date.

MAIN OUTCOMES: Case-control differences in exposures of interest were modelled for each time period and adjusted for demographic and clinical factors (ischaemic/unspecified stroke, type 2 diabetes mellitus, acute myocardial infarction, hypertension diagnosis, anti-hypertensive medication, cholesterol-lowering medication). Coefficients and interactions with time period were meta-analysed across the six databases.

RESULTS: Mean BMI (coefficient -1.16 kg/m2; 95% CI: -1.38, -0.93) and systolic BP (-2.83 mmHg; -4.49, -1.16) were lower in cases at diagnosis, and case-control differences were greater in more recent time periods, as indicated by significant case-x-time interaction and

For peer review only



For peer review only - http://bmjopen.bmj.com/site/about/guidelines.xhtml

case-x-time-squared interaction terms. Time variations in coefficients for cholesterol levels were less consistent between databases and those for diastolic BP were largely not significant.

CONCLUSION: Routine clinical data show emerging divergence in levels of BMI and SBP prior to the diagnosis of dementia but less evidence for DBP or total cholesterol levels. These divergences should receive at least some consideration in routine dementia risk screening, although underlying mechanisms still require further investigation.

Keywords: Dementia; blood pressure; body mass index; serum cholesterol; electronic health records; vascular risk factor trajectories.

For peer review only



For peer review only - http://bmjopen.bmj.com/site/about/guidelines.xhtml

## Strengths and limitations of this study

1. We have investigated associations of blood pressure, body mass index and cholesterol levels with dementia and the time-dependency of such observations, using what we believe to be currently the largest confederation of health records data.

2. There are advantages in the generalisability of routine data and in patient populations who are less subject to participation selection than research cohorts.

3. The use of multiple data resources allowed the consistency of associations to be evaluated, as it is particularly important for clinical 'big data' where large sample sizes result in even very minor associations being identified as statistically significant, and where traditional heterogeneity statistics from meta-analyses are similarly uninformative.

4. The limitations of the study are those that typify routine administrative rather than research data, in that measurements were limited to those recorded in routine practice which could be harmonised across the six databases, and the stage of dementia at diagnosis was not known.

5. While the large samples will have reduced the impact of missing data on statistical power, potential bias arising from non-random missingness cannot be excluded, although we feel it is unlikely to account for the findings of interest.

For peer review only



For peer review only - http://bmjopen.bmj.com/site/about/guidelines.xhtml

# INTRODUCTION

Vascular risk factors are widely recognised as important in the aetiology of dementia; however, observed associations depend on the timing of measurement. For example, higher blood pressure (particularly systolic blood pressure; SBP) in mid-life is a predictor of dementia 10-20 years later [1,2,3], but people with dementia tend to have lower blood pressure at the time of the clinical onset than age-matched controls [1], accounted for by an exaggerated decline in systolic blood pressure prior to the onset of dementia [4,5,6]. Similar associations have been observed for body mass index (BMI), with mid-life obesity associated with increased risk of dementia [7,8], but dementia clinical onset associated with lower contemporaneous BMI [9] and preceded by accelerated weight loss [10]. Finally, higher mid-life cholesterol has been found to predict dementia in some studies [11], and a decline in cholesterol levels prior to dementia onset has been observed in others [12,13].

These findings have arisen almost exclusively from specifically recruited research cohorts followed over long periods – advantageous in many respects but potentially subject to selection, and therefore limited generalisability. The parameters of interest (BP, BMI, cholesterol levels) are routinely measured in clinical practice, but there has been no investigation of their associations with dementia and the time-dependency of such observations. We sought to pursue this in what we believe to be currently the largest confederation of health records data – that enabled by the European Medical Information Framework Innovative Medicines Initiative (EMIF-IMI) within which an Alzheimer's Disease (EMIF-AD) consortium was developed. The objective of the study reported here was to compare levels of the exposures of interest between dementia cases and controls at multiple time intervals prior to dementia onset, and to test time interval as a modifying factor for these associations. The specific hypothesis was that BMI, systolic and diastolic blood



For peer review only - http://bmjopen.bmj.com/site/about/guidelines.xhtml

For peer review only

pressure and serum total cholesterol would be lower in people with newly diagnosed dementia than in controls, and that these differences would have widened closer to the diagnosis date. In this study we used data from several European national or regional databases – both primary care general practitioner records and hospital based databases – containing a total number of over 22.5 million patients who used mainly public/ national health services.

For peer review only



For peer review only - http://bmjopen.bmj.com/site/about/guidelines.xhtml

# METHODS

## Data resources

EMIF-IMI worked with several electronic health record (EHR) resources to render data available for analysis with appropriately robust data security and governance. EMIF datasets participating in a recent investigation of incidence and prevalence of diagnosed dementia were estimated to contain information on 182,776 people with a dementia diagnosis, with around 1,000,000 person-years of data prior to the diagnosis and 400,000 person-years after the diagnosis [14]. These resources included three primary care databases from the Netherlands, Italy and United Kingdom (Integrated Primary Care Information [IPCI], Health Search Database [HSD] and The Health Improvement Network [THIN] respectively) and two hospital catchment area databases in Jutland and Barcelona (Aarhus University Hospital Database [AUH] and Information System of Parc de Salut Mar [IMIM-UPF] respectively). In this study, the 'Information System for Research in Primary Care [SIDIAP], containing Catalan primary care records) was added as a new database. The final assembled data resource included a total 291,780 patients with a dementia diagnosis across the six databases.

The THIN database contains data from participating UK primary care services with records from 1990 on around 9 million patients in total and 4 million receiving active care at a given time. Demographic profiles have been found to be similar to national estimates although THIN contains fewer people aged under 25 years; condition prevalence has also been found to be comparable[15]. The IPCI database is drawn from primary care records in Netherlands since 1990 on around 2.6 million patients in total, and 1.4 million receiving active care; its data are drawn from what has been found to be a representative sample (n=750) of general practitioners (GPs) in the Netherlands [16]. AUH is a regional record linkage system in Aarhus, Denmark containing both inpatient and outpatient records since 1989 with 1.8



For peer review only - http://bmjopen.bmj.com/site/about/guidelines.xhtml

million active patients, drawing data from different public bodies and including all ages [17]. IMIM-UPF contains hospital data from the Barcelona region with 1.6 million active patients and including all hospital admissions. HSD contains primary care records in Italy with nearly 2.3 million patient records from more than 800 participating primary care services throughout Italy: a longitudinal observational database that has been found to be representative of the general Italian population, established in 1998 by the Italian College of General Practitioners [18]. SIDIAP contains primary care records from Catalonia in Spain with nearly 6 million patient records from family paediatricians and general practitioner medical records and data from all ages [17].

The European Medical Information Framework (EMIF) project, funded through the IMI programme (Innovative Medicines Initiative Joint Undertaking under Grant Agreement No. 115372), had designed and implemented a federated platform to connect health data from a variety of sources across Europe, to facilitate large scale clinical and life sciences research. It enabled approved users to analyse securely multiple, diverse, data via a single portal, thereby mediating research opportunities across a large quantity of research data. EMIF developed a code of practice (ECoP) to ensure the privacy protection of data subjects, protect the interests of data sharing parties, comply with legislation and various organisational policies on data protection, uphold best practices in the protection of personal privacy and information governance, and eventually promote these best practices more widely. EMIF convened an Ethics Advisory Board (EAB), to provide feedback on its approach, platform, and the EcoP [19] and all use of the data resources described was carried out with full approval of the data owners and thus complied with pre-existing governance specifications for secondary analysis.

This study created an age- and sex-matched case control study, comparing people with and without dementia on prior exposures of interest over the preceding 12 years, adjusting for relevant and available covariates, and investigating time interval as a modifying factor.



For peer review only - http://bmjopen.bmj.com/site/about/guidelines.xhtml

For peer review only

**Population**

Cases were defined on the basis of a diagnosis of dementia and included all assigned dementia diagnoses of any type (inclusion criterion) and the index date was set as that on which the first dementia diagnosis was recorded. The definition of dementia was extensively developed and harmonised across the EMIF-AD consortium, taking into account the different coding systems used, as previously described in detail including supplementary data on code lists applied [14]. For each case, at the index date of first dementia diagnosis, 100 controls were drawn randomly from the same EHR, matched to the case on gender and age (+/- 2 years) at that index date, having excluded any dementia at or prior to that date. When there were not enough controls, one control was allowed to be used for several cases. Both cases and controls were restricted to people aged 50 years or over at the index date.

**Exposures**

The following measures were extracted whenever recorded within each dataset: BMI (kg/m$^2$), systolic BP (SBP; mmHg), diastolic BP (DBP; mmHg), and serum total cholesterol (mmol/l). BMI was extracted where recorded, or was calculated from recorded body weight and height data. Levels for each of these variables were defined within six 2-year time intervals over the 12 years prior to the index date, taking mean values if multiple measures were present for an individual within each interval. Percentages of missing data points were compared between cases and controls, and by age, sex and comorbidities.

**Covariates**



For peer review only - http://bmjopen.bmj.com/site/about/guidelines.xhtml

Data on age at index date and gender were extracted. In addition, the following binary variables were generated based on any recorded diagnosis/treatment prior to the index date: ischaemic/unspecified stroke, type 2 diabetes mellitus (diagnosis or antidiabetic medication), acute myocardial infarction, hypertension diagnosis, use of any anti-hypertensive medication and use of any cholesterol-lowering medication. Hypertension diagnosis was considered as a relevant covariate as patients with sustained hypertension in midlife have an increased risk for dementia regardless of their BP during late life [20] and because the interest was on changes in the BP relationship over time, which was not considered to be coterminous with a hypertension diagnosis. Medication information was extracted from databases in which it was available from recorded prescriptions or dispensations; disorders were defined according to pre-existing harmonised data extraction criteria used across the EMIF-IMI consortia [21].

**Statistical analysis**

The total pool of cases and controls were first described in terms of demographic and clinical covariates, followed by a descriptive analysis of data availability on the exposures of interest.

General Linear Model (GLM) analyses were used for each database and for each of the four parameters of interest, to model case-control differences in mean exposure levels separately for each comparison time period and to adjust for covariates. Importantly, each time period comparison was treated as a separate analysis for case-control sets with data present in that time period and covariate adjustments for each comparison, rather than in terms of repeated observations within individuals. The exposures here were modelled as dependent variables, with main independent variables being case-control status, time category, interaction between case-control status and time category, age at index date, sex, the four vascular disorders described above, and stratum variable. Based on plots of unadjusted mean differences by time



For peer review only - http://bmjopen.bmj.com/site/about/guidelines.xhtml

period, linear time terms were used for DBP as a dependent variable while quadratic time terms were added to models of BMI, SBP and total cholesterol.

Having finalised models for each of the four parameters (BMI, SBP, DBP, total cholesterol) in each EHR dataset, the GLM coefficients for each dataset were pooled in a meta-analysis for each parameter. Random effects were assumed when coefficients were pooled. In secondary analyses, adjusted meta-analysed coefficients for SBP, DBP and total cholesterol were stratified as appropriate according to previously received antihypertensive or lipid lowering medication in cases and controls. The significance level for all tests was defined as a p-value $\leq 0.05$. Analyses were carried out using IBM SPSS Statistics version 20.0.

**Patient and public involvement**

The was no specific patient or public involvement in the design or interpretation of this particular study**.**

For peer review only



For peer review only - http://bmjopen.bmj.com/site/about/guidelines.xhtml

## RESULTS

Overall, there were 291,780 dementia cases (ranging from 3,433 cases in IMIM-UPF to 139,083 cases in THIN) and 29,170,549 controls made available for analysis from all six EHR databases. As described in Table 1, age and sex were effectively matched in cases and controls. Higher proportions of cases had a history of diabetes, acute myocardial infarction and stroke. Slightly higher proportions of cases had been prescribed antihypertensive, as well as lipid lowering, medication than controls; however, prevalence of recorded hypertension did not differ consistently between cases than controls across the databases. Descriptive data on mean vascular risk factor levels by case/control status, time period and database are provided in Supplementary Table 1, missing data percentages by year and case status in Supplementary Table 2, and missing data by covariates in Supplementary Table 3. Missing data were more prominent for less recent time periods; for time periods over 2 years before the index date, missing data were more common in cases than controls, but more common in controls than cases for the 0-2 year time period; missing data were least common for SBP/DBP and most common for BMI. Mean numbers of missing data points were not found to vary consistently by age or sex, although missing data were consistently below mean levels in those with hypertension and above mean levels in those with stroke. Supplementary table 2, summarises proportions of patients with missing data for each vascular risk factor in each database. In summary, AUH had limited BMI data, with no values prior to 6 years before the index date and no data for SBP and DBP. For HSD, 94% of BMI data were missing in the 10-12 year window before the index date, improving to 78% by the 0-2 year window and similar trends were observed for BMI data in the IPCI database, with 93% missing at 10-12 years and 67% missing data at 0-2 years before the index date. In the SIDIAP database there were no BMI, blood pressure or total cholesterol data at 10-12 years before the index date. Generally, compared with other databases, there were fewer missing data in the THIN



For peer review only - http://bmjopen.bmj.com/site/about/guidelines.xhtml

database for all risk factors. IMM-UPF did not have blood pressure data, and had no BMI data at 10-12 years before the index date.

Figure 1 displays case-control differences in vascular risk factor exposures of interest by time and database. For BMI, the largest decline in meta-analysed case-control differences was observed between 2-4 years and 0-2 years before the index diagnosis of dementia. For SBP, the meta-analysed case-control difference increased from 8-10 years to 4-6 years and then declined steeply from 2-4 years to 0-2 years. For DBP, the largest meta-analysed case-control differences were observed in the 2-4 year and 0-2 year periods before the diagnosis of dementia. For total serum cholesterol levels, the meta-analysed case-control difference increased from 10-12 years to 6-8 years before the diagnosis of dementia and then declined up till the diagnosis of dementia with a slightly large decline just before diagnosis of dementia. For all exposures apart from DBP, cases had lower values compared with controls (negative case coefficients), all four exposures decreased in later time periods (negative time coefficients) and this was more marked for cases compared to controls (negative case-x-time interaction terms) (Table 2). Quadratic terms were also significant and negative in value for BMI and SBP, reflecting the curvilinear relationships displayed in Figure 1, while those for total cholesterol comprised a positive time-squared term and a negative case-x-time-squared interaction, again reflecting the curvilinear visualised relationship. Following adjustment for covariates, case-x-time and case-x-time-squared interaction terms were reduced modestly for BMI and SBP but retained statistical significance. No significant case or case-x-time terms were observed for DBP. For total cholesterol a negative-value case-x-time interaction term persisted, but there was no longer a significant case-x-time-squared interaction.

Following additional stratification of these adjusted models by medication use (Table 3), SBP coefficients were not markedly different (overlapping confidence intervals), although those for case status and the case-x-time interaction were stronger in the subgroups who were not



For peer review only - http://bmjopen.bmj.com/site/about/guidelines.xhtml

receiving antihypertensive medication; the (negative value) case coefficient for DBP was also stronger in that subgroup. Likewise, case and case-x-time coefficients for total cholesterol levels were stronger in those not receiving lipid lowering medication, although confidence intervals overlapped.

For peer review only



For peer review only - http://bmjopen.bmj.com/site/about/guidelines.xhtml

**DISCUSSION**

In this large study spanning 12 years, we compared people with/without a dementia diagnosis and investigated previously recorded levels of BMI, SBP, DBP, and total serum cholesterol. In summary, BMI, SBP and total cholesterol levels were lower overall in cases with dementia than controls and the difference was most marked closest to the point of diagnosis. This time-dependency of the associations was not accounted for by age, sex or recorded vascular disorders, and case-control differences in SBP and total cholesterol did not appear to be modified by antihypertensive or lipid lowering treatment respectively. There was little evidence for associations with DBP after adjustment, and between-database heterogeneity appeared high for associations with total cholesterol, unlike the relative consistency observed with BMI and SBP. As described, while dementia is generally found to be associated with higher levels of vascular risk factors measured 10-20 years before its clinical onset in prospective epidemiological studies, associations are often found to be reversed when investigated closer to time of this clinical onset [22]. The extent to which these associations can be observed in more healthcare data remains to be established, despite opportunities for assessing this over large, 'real world' populations.

Considering BMI, prospective studies with decades follow-up [23] have suggested that midlife obesity is a risk factor for dementia [24], but this association is attenuated or reversed in older age [25], and exaggerated weight loss precedes the clinical onset of dementia [26], described for periods of 2 to 4 years [27], 5 years [28], or up to 10 years [29] before dementia onset. Our findings of lower SBP in cases at the time of diagnosis, and a preceding widening difference over time, are also consistent with epidemiological research, as is the lack of association with DBP: high mid-life SBP is a risk factor for dementia incidence 10-20 years



For peer review only - http://bmjopen.bmj.com/site/about/guidelines.xhtml

later [5, 2, 3], while SBP at dementia clinical onset tends to be lower than controls [5, 30], and exaggerated decline in SBP prior to dementia has been described in Swedish [4, 31] and Japanese American [6] cohorts, as well as during the course of dementia [32]. Although case and case-x-time coefficients were statistically significant for total cholesterol levels, similar to those for BMI and SBP, visual inspection (Figure 1) indicated considerably more inconsistency between databases and we feel that conclusions are less strong in this respect. The relationship between cholesterol level and dementia has been less clear in epidemiological research [33], although tends to support associations with high total cholesterol mid-life but not in late-life. Declining cholesterol levels precede dementia [12, 13], although without the later acceleration described for BMI and SBP, and not observed in all studies [34].

Assuming that the widening gaps in exposure levels between cases and controls in our data reflect dementia-associated decline in these factors, underlying causal pathways may vary. Weight loss might be secondary to early neurodegeneration, reflecting a combination of pre-dementia apathy, loss of initiative, and reduced olfactory function [29]. Cholesterol decline in one study was observed to occur over 10 years before dementia clinical onset, followed by a parallel trajectory [13], so could conceivably be a marker for another risk factor, such as an episode of infection or inflammation resulting in both the decline in cholesterol and increased later risk of dementia. While declining blood pressure might be secondary to early neurodegeneration, it might also represent a risk factor in itself, through reduced cerebral perfusion and watershed ischaemia/infarction, and/or might be a consequence of previous untreated hypertension. The role of antihypertensive treatment in accounting for or modifying pre-dementia blood pressure decline has been controversial. In one cohort, the exaggerated dementia-associated decline in SBP was found to be restricted to people who had not received antihypertensive agents [6], and associations between dementia and lower



For peer review only - http://bmjopen.bmj.com/site/about/guidelines.xhtml

contemporaneous blood pressure were found to be weak or absent in populations from low- and middle-income countries where hypertension is uncommon [35]. However, other cohorts have found stronger contemporaneous associations between dementia and lower blood pressure in people receiving antihypertensives [36] or no modification of later SBP decline [31]. In our databases, the coefficients of interest (i.e. for case and case-x-time) were stronger in the subgroup who had not received antihypertensive treatment, but differences were not substantial and confidence intervals overlapped. The same was true for associations with cholesterol levels, which were also marginally stronger in the subgroup who had not received lipid lowering treatment, although no attempt was made to factor in treatment differences in lipid-lowering effectiveness.

Our study has several strengths. We believe that the data resource used for this analysis is the largest to date by a considerable margin, illustrating the potential value of routine healthcare data and increasing accessibility of electronic health records for research use within robust governance frameworks such as that developed by the EMIF-IMI consortium. In addition, there are advantages in the generalisability of routine data and in patient populations who are less subject to participation selection than research cohorts. Finally, the use of multiple data resources allowed the consistency of associations to be evaluated. This is particularly important for clinical 'big data' where large sample sizes result in even very minor associations being identified as statistically significant, and where traditional heterogeneity statistics from meta-analyses are similarly uninformative; this is exemplified in the visualisation of associations with DBP and total cholesterol (Figure 1) where reported analyses from single databases might have given rise to conclusions which are not consistent across others.



For peer review only - http://bmjopen.bmj.com/site/about/guidelines.xhtml

In previous research, there has been some suggestion of an age-dependent effect of blood pressure on Alzheimer disease, although the evidence is relatively weak: there was a suggestion that midlife diastolic, but not midlife systolic, hypertension in midlife may increase risk of incident Alzheimer disease and a suggestion that elevated late-life BP may actually be beneficial [37]. However, the same systematic review [37] and meta-analysis of prospective epidemiologic research, did not provide clear evidence for a relationship between blood pressure and incidence of Alzheimer disease. On the other hand, [38] found higher SBP among patients with vascular dementia (OR per 10 mmHg: 1.33). Several well-established longitudinal studies have reported on the relationships between the trajectory of change over time in levels of blood pressure, cholesterol and BMI and incident all-cause dementia, with some additionally reporting on Alzheimer's disease, vascular dementia and MCI [39]. Authors found that the results for Alzheimer's disease, vascular dementia and MCI were similar but with fewer data points, with those who went on to develop all-cause dementia showing a greater increase followed by a sharper decrease in blood pressure and/or BMI before a positive diagnosis.

In a collaborative study of over 1.3 million adults from Europe, the United States, and Asia, higher BMI was associated with increased dementia risk when weight was measured >20 years before dementia diagnosis [40], but this association was reversed when BMI was assessed <10 years before dementia diagnosis and this finding is consistent with our study as well as a systematics review [41]. Furthermore, comparisons of risk factors for dementia, AD, and VaD, in developed and developing world regions were carried out in a review by Kalaria in 2010 which concluded that there was a high risk effect of factors such as vascular diseases and smoking for incidence of dementia in developed regions (North America, Europe, Japan) as well as in Asia (China, Guam, India, South Korea, Taiwan). Dementia was significantly associated with reported stroke and diabetes in logistic regression models



For peer review only - http://bmjopen.bmj.com/site/about/guidelines.xhtml

adjusted for sociodemographic status and other vascular risk factors (OR (95%CI) 4.40 (2.70 to 7.19) and 1.56 (1.20 to 2.03), respectively) in a national survey of older people in Trinidad [42].

Information on education level was not available in these routine healthcare data resources. On the one hand, low educational achievement has been shown to be a robust risk factor for dementia[43]; on the other hand, intellectually stimulating, socially engaging, or physical activities might lower the risk of dementia [44]. The situation is not different in developing countries, where surveys have consistently identified low education as a risk factor for dementia [45]. However, in some communities, low literacy is often linked to poverty or lower socioeconomic status, which is also associated with poorer health, lower access to health care, and increased risk of dementia [45]. While it may well be the case that lower education confers both a higher risk of dementia and a higher risk of adverse vascular risk factors, it is less clear whether there should be any association between education and the changes over time in relationships between vascular risk factors and dementia, described in this study.

The limitations of the study are those that typify routine administrative rather than research data. Measurements were limited to those recorded in routine practice which could be harmonised across the six databases, bearing in mind the different clinical coding systems used. For this reason, and as previously described [14] dementia was defined as a composite outcome with no attempt to identify subtypes which are less consistently ascertained in routine clinical practice. Covariates were limited to demographic factors, common disorders, and specific medication groups of interest, and did not include measures of socioeconomic status or lifestyle factors such as smoking status, diet, or physical activity. Missing data are also an important consideration. While the large samples will have reduced the impact of missing data on statistical power, potential bias arising from non-random missingness cannot



For peer review only - http://bmjopen.bmj.com/site/about/guidelines.xhtml

be excluded, although we feel it is unlikely to account for the findings of interest. As displayed in Supplementary Table 2, the strongest influence on data loss was time, with more missing data in earlier time periods, as would be expected in clinical samples (where routine exposure checks will be more common in older age groups). Therefore, case-control differences at earliest time points should be viewed with caution. Furthermore, two databases, AUH and IMIM-UPF, did not contribute blood pressure data and IMIM-UPF and HSD had high proportion of missing data for BMI throughout the study period. However, it is hard to envisage how differential missing data could account for lower exposure levels in future cases, or for the widening of case-control differences over time (since this was not observed for all exposures of interest). Furthermore, influences of age, sex and comorbidity on missingness were relatively minor, apart from understandable higher recording in monitored conditions such as hypertension. Finally, although missing data were more common in cases than controls for most time periods, these differences were not substantial. Consideration was given to missing data when analyses were planned; however, the limited level of covariate ascertainment and potential non-random missingness were felt to preclude imputation approaches.

An important consideration with dementia diagnoses in routine healthcare data from non-specialist services is that there is minimal or no information on the stage/severity of dementia at diagnosis, because of the absence of structured data on cognitive function. While some people will have received a dementia diagnosis early in the course of the condition, others may receive this after several years of significant symptoms. For example, the length of time between dementia symptoms being noticed and diagnosis of dementia has been estimated to vary in Europe from 1.61 years in Italy to 2.49 years in Scotland and 2.57 years in Netherlands [46]. Therefore, comparisons are limited with epidemiological cohorts using dementia screening protocols. At least some of the widening difference in exposure levels



For peer review only - http://bmjopen.bmj.com/site/about/guidelines.xhtml

between cases and controls may therefore have occurred during times when dementia was evident but undiagnosed. Finally, our analyses did not analyse exposure trajectories within individuals, which is the standard approach in epidemiological cohorts. Instead we investigated case-control differences in previously recorded exposures at different times, hypothesising that the dementia-associated trajectories previously reported for these factors would manifest in variation of case-control differences across time periods, and hence case-x-time interaction in the statistical models.

## Conclusion

In summary, considering our observations at group level, there are clear potential implications for clinical services which require further evaluation. For example, there might be good reason for individual trajectories to be more effectively monitored (for example, BMI or SBP change), possibly enhanced by EHR systems that could highlight changes over time and the need for more frequent assessment or supplementary cognitive assessment for earlier risk-identification. In addition, regardless of causation, the potential consequences of low BMI/SBP as comorbidities in dementia have yet to be fully clarified (e.g. as risk factors for falls or functional decline) which again might have implications for future routine monitoring. In examining the patterns and trajectories of the established risk factors for cognitive decline and dementia, this study adopted a relatively broad approach to an emerging area of enquiry. Our study focused on three risk factors of interest that were envisaged to be most available (blood pressure, cholesterol and BMI); however, future work could feasibly include greater numbers of risk factors and the interaction between different individual changes in status (for example the combination of changes in blood pressure and BMI), which could eventually lead to more personalised risk assessments and targeted interventions early in the asymptomatic, prodromal phase of cognitive decline and dementia.



For peer review only - http://bmjopen.bmj.com/site/about/guidelines.xhtml

**Figure 1: Mean differences between cases and controls in vascular risk factor exposures of interest by database and time prior to index date**

For peer review only



For peer review only - http://bmjopen.bmj.com/site/about/guidelines.xhtml

**Table 1: Characteristics of component databases: demographic factors and comorbidities**

| | AUH | | HSD | | IPCI | | SIDIAP | | THIN | | IMIM-UPF | |
|---|---|---|---|---|---|---|---|---|---|---|---|---|
| | Case | Control | Case | Control | Case | Control | Case | Control | Case | Control | Case | Control |
| Number | 4,756 | 475,319 | 21,465 | 2,145,779 | 14,039 | 1,401,114 | 109,004 | 10,900,377 | 139,083 | 13,907,094 | 3,433 | 340,866 |
| Mean (SD) age | 78.8 (9.7) | 78.7 (9.7) | 80.6 (8.3) | 80.4 (8.2) | 81.1 (8.7) | 80.9 (8.6) | 81.4 (8.1) | 81.3 (8.1) | 80.9 (8.9) | 80.6 (8.9) | 80.0 (9.6) | 79.8 (9.5) |
| Males (%) | 43.0 | 43.0 | 32.8 | 32.8 | 36.7 | 36.7 | 33.8 | 33.8 | 34.8 | 34.8 | 42.1 | 42.0 |
| **Medication use (%)** | | | | | | | | | | | | |
| Antihypertensive | 79.1 | 78.2 | 76.1 | 72.9 | 75.0 | 75.4 | 77.2 | 73.8 | 56.6 | 63.8 | n/a | n/a |
| Lipid lowering | 47.8 | 45.0 | 26.9 | 26.1 | 42.3 | 39.4 | 46.6 | 42.2 | n/a | n/a | n/a | n/a |
| **Comorbidities (%)** | | | | | | | | | | | | |
| Diabetes | 13.0 | 9.5 | 23.1 | 19.8 | 21.2 | 18.9 | 26.6 | 18.9 | 13.0 | 10.7 | 29.7 | 14.0 |
| Hypertension | 40.3 | 33.6 | 63.9 | 61.0 | 48.6 | 52.2 | 64.5 | 64.2 | 49.6 | 52.5 | 61.0 | 37.9 |
| Acute MI | 6.7 | 7.3 | 2.4 | 2.1 | 7.5 | 6.4 | 3.4 | 2.6 | 8.2 | 5.9 | 4.2 | 2.7 |
| Stroke | 13.8 | 7.8 | 8.3 | 6.5 | 12.4 | 7.7 | 12.3 | 6.9 | 10.6 | 5.7 | 12.8 | 3.9 |

n/a: Data not available



For peer review only - http://bmjopen.bmj.com/site/about/guidelines.xhtml

**Table 2: Meta-analysed unadjusted GLM coefficients (with 95% confidence intervals) across component databases for the relationships between case/control status, time period and the four outcomes of interest**

| | BMI | SBP* | DBP*$ | Cholesterol |
|---|---|---|---|---|
| **Unadjusted** | | | | |
| Intercept | 31.6 (31.6, 31.6) | 114.6 (114.5, 114.6) | 84.4 (84.4, 84.4) | 5.4(5.3, 5.5) |
| Case | -1.32 (-1.41, -1.21) | -3.25 (-3.34, -3.16) | 0.04 (-0.03, 0.11) | -0.12 (-0.20, -0.04) |
| Time | -0.11 (-0.17, -0.06) | -1.06 (-1.17, -0.98) | -0.54 (-0.61, -0.45) | -0.03 (-0.05, -0.01) |
| case-x-time | -0.27 (-0.32, -0.19) | -0.63 (-0.72, -0.57) | -0.03 (-0.06, -0.01) | -0.03 (-0.04, -0.02) |
| Time squared | -0.06 (-0.10, -0.02) | -0.13 (-0.19, -0.07) | | 0.01 (0.00, 0.02) |
| case-x-time squared | -0.02 (-0.03, -0.01) | -0.14 (-0.21, -0.10) | | -0.01 (-0.01, -0.00) |
| **Adjusted**** | | | | |
| Intercept | 31.5 (30.9, 32.2) | 117.6 (108.8, 126.5) | 87.0 (81.7, 92.3) | 5.18 (4.89, 5.50) |
| Case | -1.16 (-1.38, -0.93) | -2.83 (-4.49, -1.16) | -0.19 (-0.46, 0.08) | -0.10 (-0.17, -0.04) |
| Time | -0.13 (-0.16, -0.10) | -0.83 (-1.16, -0.50) | -0.50 (-0.57, -0.42) | -0.05 (-0.07, -0.02) |
| case-x-time | -0.18 (-0.28, -0.08) | -0.53 (-0.80, -0.26) | 0.00 (-0.06, 0.06) | -0.05 (-0.09, -0.01) |
| Time squared | -0.01 (-0.08, 0.06) | -0.01 (-0.02, 0.02) | | 0.00 (-0.02, 0.02) |
| case-x-time squared | -0.01 (-0.02, -0.00) | -0.04 (-0.05, -0.02) | | -0.01 (-0.01, 0.01) |

$ Based on linear equation; *SBP and DBP not available in AUH or IMIM-UPF; **GLM coefficients adjusted for age, sex, diabetes, hypertension, acute myocardial infarction, and stroke



For peer review only - http://bmjopen.bmj.com/site/about/guidelines.xhtml

**Table 3: Meta-analysed adjusted GLM coefficients stratified by antihypertensive treatment and lipid lowering medication**

| SBP | With antihypertensive treatment | No antihypertensive treatment |
|---|---|---|
| Intercept | 121.6 (115.5, 127.8) | 109.4 (101.4, 117.5) |
| Case | -2.44 (-3.56, -1.33) | -4.02 (-8.67, 0.63) |
| Time | -1.01 (-1.40, -0.62) | -0.14 (-0.55, 0.28) |
| case-x-time | -0.49 (-0.60, -0.32) | -0.72 (-1.54, 0.10) |
| Time squared | -0.03 (-0.04, -0.01) | -0.04 (-0.05, -0.02) |
| case-x-time squared | -0.04 (-0.05, -0.03) | -0.04 (-0.08, 0.01) |

| DBP | With antihypertensive treatment | No antihypertensive treatment |
|---|---|---|
| Intercept | 89.3 (85.8, 92.9) | 82.3 (76.1, 88.5) |
| Case | -0.05 (-0.43, 0.33) | -1.07 (-1.57, -0.56) |
| Time | -0.55 (-0.64, -0.50) | -0.15 (-0.22, -0.08) |
| case-x-time | 0.02 (-0.02, 0.07) | -0.11 (-0.29, 0.07) |

| Cholesterol | With lipid lowering drugs | No lipid lowering drugs |
|---|---|---|
| Intercept | 5.23 (5.20, 5.25) | 5.44 (5.18, 5.71) |
| Case | 0.02 (-0.01, 0.05) | -0.08 (-0.12, -0.03) |
| Time | -0.07 (-0.10, -0.04) | -0.02 (-0.06, 0.02) |
| case-x-time | -0.01 (-0.01, 0.01) | -0.03 (-0.04, -0.02) |
| Time squared | -0.03 (-0.04, -0.01) | -0.04 (-0.05, -0.02) |
| case-x-time squared | -0.01 (-0.02, 0.00) | -0.01 (-0.01, -0.00) |



For peer review only - http://bmjopen.bmj.com/site/about/guidelines.xhtml

For peer review only

## References

[1] Qiu C., Von Strauss E., Fastbom J., Winblad B., Fratiglioni L. Low blood pressure and risk of dementia in the Kungsholmen project: a 6-year follow-up study. Arch Neurol. 2003; 60: 223–228.

[2] Launer L.J., Ross G.W., Petrovitch H., Masaki K., Foley D., White L.R. et al. Midlife blood pressure and dementia: The Honolulu-Asia Aging study. Neurobiol Aging. 2000;21(1): 49–55.

[3] Kivipelto M., Helkala E.L., Laakso M.P., Hanninen T., Hallikainen M., Alhainen K. et al. (2001) Midlife vascular risk factors and Alzheimer's disease in later life: longitudinal, population based study. BMJ 322: 1447–1451.

[4] Skoog I, Lernfelt B, Landahl S, Palmertz B, Andreasson LA, Nilsson L, Persson G, Oden A, Svanborg A. 15-Year longitudinal study of blood pressure and dementia. Lancet. 1996; 347:1141–1145.

[5] Qiu C, von Strauss E, Winblad B, Fratiglioni L. (2004). Decline in blood pressure over time and risk of dementia: a longitudinal study from the Kungsholmen project. Stroke. 35(8):1810-5.

[6] Stewart R, Xue QL, Masaki K, Petrovitch H, Ross GW, White LR, Launer LJ. Change in blood pressure and incident dementia: a 32-year prospective study. Hypertension. 2009; 54:233–240.

[7] Gustafson DR, Bäckman K, Joas E, Waern M, Östling S, Guo X, Skoog I. 37 years of body mass index and dementia: observations from the Prospective Population Study of Women in Gothenburg, Sweden. J Alzheimer Dis. 2012;28:163–171.



For peer review only - http://bmjopen.bmj.com/site/about/guidelines.xhtml

[8] Whitmer R.A., Gustafson D.R., Barrett-Connor E., Haan M.N., Gunderson E.P., Yaffe K. Central obesity and increased risk of dementia more than three decades later. Neurology. 2008;71:1057–1064.

[9] Nourhashemi F, Deschamps V, Larrieu S, et al. Body mass index and incidence of dementia: the PAQUID study. Neurology . 2003.;60(1):117-9.

[10] Johnson DK, Wilkins CH, Morris JC. Accelerated Weight Loss May Precede Diagnosis in Alzheimer Disease. Arch Neurol. 2006;63(9):1312–1317.

[11] Notkola IL, Sulkava R, Pekkanen J, et al. Serum total cholesterol, apolipoprotein E epsilon 4 allele, and Alzheimer's disease. Neuroepidemiology. 1998;17:14–20.

[12] Mielke, M. M., Zandi, P. P., Shao, H., Waern, M., Östling, S., Guo, X., Björkelund, C., Lissner, L., Skoog, I., … Gustafson, D. R. The 32-year relationship between cholesterol and dementia from midlife to late life. Neurology. 2010;75(21): 1888-95.

[13] Stewart R, White LR, Xue QL, Launer LJ. Twenty-six-year change in total cholesterol levels and incident dementia: the Honolulu-Asia Aging Study. Arch Neurol 2007;64:103–107.

[14] Perera G., Pedersen L., Ansel D., Alexander M., Arrighi H.M., Avillach P., Foskett N., Gini R., Gordon M.F., Gungabissoon U., Mayer M.A., Novak G., Rijnbeek P., Trifirò G., van der Lei J., Visser P.J., Stewart R.(2018) Dementia prevalence and incidence in a federation of European Electronic Health Record databases: The European Medical Informatics Framework resource. Alzheimers Dement. 2018 Feb;14(2):130-139.

[15] Blak BT, Thompson M, Dattani H, Bourke A. Generalisability of The Health Improvement Network (THIN) database: demographics, chronic disease prevalence and mortality rates. Inform Prim Care. 2011;19(4):251-255.



For peer review only - http://bmjopen.bmj.com/site/about/guidelines.xhtml

[16] Rijnbeek, PR, Voss, EA, de Wilde, M, Schuemie, M, van der Lei, J. Conversion of a Dutch general practitioner's database to the OMOP-CDM: The IPCI Database. OHDSI Symposium, Bethesda, MD; Oct. 2017.

[17 ]Sturkenboom M, Braeye T, van der Aa L, et al. ADVANCE database characterisation and fit for purpose assessment for multi-country studies on the coverage, benefits and risks of pertussis vaccinations [published online ahead of print, 2020 Feb 12]. Vaccine. 2020;S0264-410X(20)30153-5.

[18] Filippi A, Vanuzzo D, Bignamini AA, Sessa E, Brignoli O, et al. (2005) Computerized general practice databases provide quick and cost-effective information on the prevalence of angina pectoris. Italian Heart Journal: Official Journal of the Italian Federation of Cardiology 6: 49–51.

[19] Floridi, L., Luetge, C., Pagallo, U. et al. Key Ethical Challenges in the European Medical Information Framework. Minds & Machines 29, 355–371 (2019).

[20] Walker KA, Sharrett AR, Wu A, et al. Association of Midlife to Late-Life Blood Pressure Patterns With Incident Dementia. JAMA. 2019;322(6):535-545.

[21] Roberto G, Leal I, Sattar N, Loomis AK, Avillach P, Egger P, van Wijngaarden R, Ansell D, Reisberg S, Tammesoo ML, Alavere H, Pasqua A, Pedersen L, Cunningham J, Tramontan L, Mayer MA, Herings R, Coloma P, Lapi F, Sturkenboom M, van der Lei J, Schuemie MJ, Rijnbeek P, Gini R. (2016). Identifying Cases of Type 2 Diabetes in Heterogeneous Data Sources: Strategy from the EMIF Project. 11(8):e0160648

[22] Verghese J, Lipton RB, Hall CB, et al. Low blood pressure and the risk of dementia in very old individuals. Neurology. 2003;61:1667–72.



For peer review only - http://bmjopen.bmj.com/site/about/guidelines.xhtml

[23] Singh-Manoux, A., Fayosse, A., Sabia, S., Tabak, A., Shipley, M., Dugravot, A., & Kivimäki, M. Clinical, socioeconomic, and behavioural factors at age 50 years and risk of cardiometabolic multimorbidity and mortality: A cohort study. PLoS Med. 2018; 15(5): e1002571.

[24] Chuang Y.F., An Y., Bilgel M., Wong D.F., Troncoso J.C., O'Brien R.J. Midlife adiposity predicts earlier onset of Alzheimer's dementia, neuropathology and presymptomatic cerebral amyloid accumulation. Mol Psychiatry. 2016;21:910–915.

[25] Luchsinger J.A., Patel B., Tang M.X., Schupf N., Mayeux R. Measures of adiposity and dementia risk in elderly persons. Arch Neurol. 2007;64:392–398.

[26] Hughes T.F., Borenstein A.R., Schofield E., Wu Y., Larson E.B. Association between late-life body mass index and dementia: the Kame Project. Neurology. 2009;72:1741–1746.

[27] Stewart R., Masaki K., Xue Q.L., Peila R., Petrovitch H., White L.R. A 32-year prospective study of change in body weight and incident dementia: the Honolulu-Asia Aging Study. Arch Neurol. 2005;62:55–60.

[28] Buchman A.S., Wilson R.S., Bienias J.L., Shah R.C., Evans D.A., Bennett D.A. Change in body mass index and risk of incident Alzheimer disease. Neurology. 2005; 65:892–897.

[29] Knopman D.S., Edland S.D., Cha R.H., Petersen R.C., Rocca W.A. Incident dementia in women is preceded by weight loss by at least a decade. Neurology. 2007;69:739–746.

[30] Guo X, Pantoni L, Simoni M, Bengtsson C, Bjorkelund C, Lissner L, Gustafson D, Skoog I. Blood pressure components and changes in relation to white matter lesions: a 32-year prospective population study. Hypertension. 2009;54:57–62.

For peer review only



For peer review only - http://bmjopen.bmj.com/site/about/guidelines.xhtml

[31] Joas E., Bäckman K., Gustafson D., Ostling S., Waern M., Guo X., Skoog I. Blood pressure trajectories from midlife to late life in relation to dementia in women followed for 37 years. Hypertension. 2012;59:796-801.

[32] Hanon O, Latour F, Seux M, Lenoir H, Forette F, Rigaud A, Real F. Evolution of blood pressure in patients with Alzheimer's disease: a one year survey of a French cohort. J Nutr Health Aging. 2005;9:106–111.

[33] Anstey, K. J., Ashby-Mitchell, K., & Peters, R. Updating the Evidence on the Association between Serum Cholesterol and Risk of Late-Life Dementia: Review and Meta-Analysis. Journal of Alzheimer's disease : JAD. 2017; 56(1): 215-228.

[34] Wagner M, Helmer C, Tzourio C, Berr C, Proust-Lima C, Samieri C. Evaluation of the Concurrent Trajectories of Cardiometabolic Risk Factors in the 14 Years Before Dementia. JAMA Psychiatry. 2018;75(10):1033–1042.

[35] Albanese, E., Lombardo, F. L., Prince, M. J., & Stewart, R. Dementia and lower blood pressure in Latin America, India, and China: a 10/66 cross-cohort study. Neurology. 2013; 81(3): 228-35.

[36] Ruitenberg A, Skoog I, Ott A, Aevarsson O, Witteman J, C, M, Lernfelt B, van Harskamp F, Hofman A, Breteler M, M, B: Blood Pressure and Risk of Dementia: Results from the Rotterdam Study and the Gothenburg H-70 Study. Dement Geriatr Cogn Disord. 2001;12:33-3

[37] Power, M. C., Weuve, J., Gagne, J. J., McQueen, M. B., Viswanathan, A., & Blacker, D. (2011). The association between blood pressure and incident Alzheimer disease: a systematic review and meta-analysis. Epidemiology (Cambridge, Mass.), 22(5), 646–659.

For peer review only



For peer review only - http://bmjopen.bmj.com/site/about/guidelines.xhtml

[38] Yamada M., Kasagi F., Sasaki H., Masunari N., Mimori Y., Suzuki G. (2003) Association between dementia and midlife risk factors: The Radiation Effects Research Foundation Adult Health Study. J Am Geriatr Soc 51: 410–414.

[39] Peters R, Peters J, Booth A, Anstey KJ. Trajectory of blood pressure, body mass index, cholesterol and incident dementia: systematic review. Br J Psychiatry. 2020;216(1):16-28.

[40] Kivimäki, M., Luukkonen, R., Batty, G. D., Ferrie, J. E., Pentti, J., Nyberg, S. T., Shipley, M. J., Alfredsson, L., Fransson, E. I., Goldberg, M., Knutsson, A., Koskenvuo, M., Kuosma, E., Nordin, M., Suominen, S. B., Theorell, T., Vuoksimaa, E., Westerholm, P., Westerlund, H., Zins, M., … Jokela, M. (2018). Body mass index and risk of dementia: Analysis of individual-level data from 1.3 million individuals. Alzheimer's & dementia : the journal of the Alzheimer's Association, 14(5), 601–609.

[41] Pedditizi E., Peters R., Beckett N. The risk of overweight/obesity in mid-life and late life for the development of dementia: a systematic review and meta-analysis of longitudinal studies. Age Ageing. 2016;45:14–21.

[42] Davis G, Baboolal N, Mc Rae A, et alDementia prevalence in a population at high vascular risk: the Trinidad national survey of ageing and cognitionBMJ Open 2018;8:e018288.

[43] Borenstein AR, Copenhaver CI, Mortimer JA. Early-life risk factors for Alzheimer disease. Alzheimer Dis Assoc Disord. 2006;2:63–72.

[44] Fratiglioni L, Paillard-Borg S, Winblad B. An active and socially integrated lifestyle in late life might protect against dementia. Lancet Neurol. 2004;3(6):343-353.

[45] Kalaria, R. N., Maestre, G. E., Arizaga, R., Friedland, R. P., Galasko, D., Hall, K., Luchsinger, J. A., Ogunniyi, A., Perry, E. K., Potocnik, F., Prince, M., Stewart, R., Wimo, A.,



For peer review only - http://bmjopen.bmj.com/site/about/guidelines.xhtml

Zhang, Z. X., Antuono, P., & World Federation of Neurology Dementia Research Group (2008). Alzheimer's disease and vascular dementia in developing countries: prevalence, management, and risk factors. The Lancet. Neurology, 7(9), 812–826.

[46] Woods, B., Arosio, F., Diaz, A., Gove, D., Holmerová, I., Kinnaird, L., Mátlová, M., Okkonen, E., Possenti, M., Roberts, J., Salmi, A., van den Buuse, S., Werkman, W., & Georges, J. (2019). Timely diagnosis of dementia? Family carers' experiences in 5 European countries. International journal of geriatric psychiatry, 34(1), 114–121.

For peer review only



For peer review only - http://bmjopen.bmj.com/site/about/guidelines.xhtml

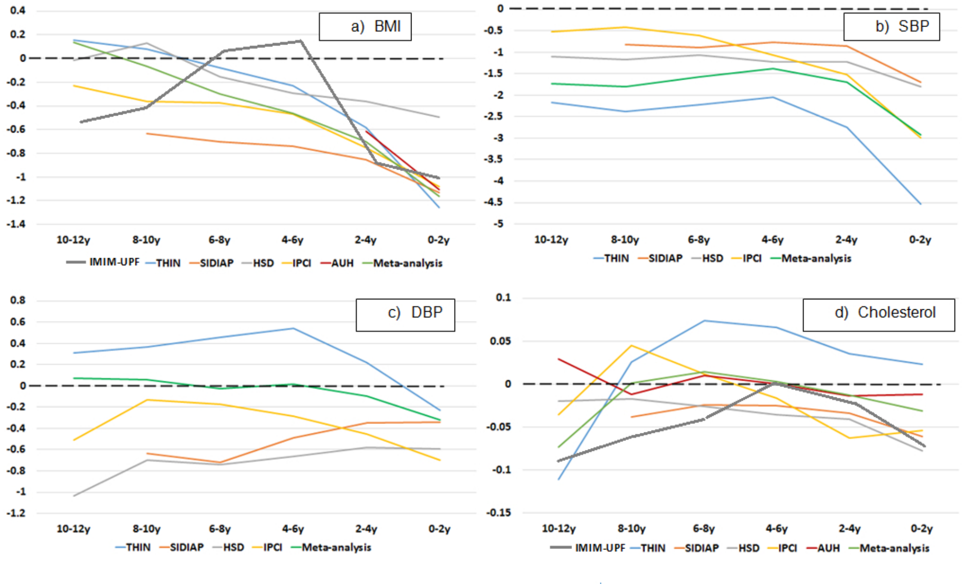


Figure 1: Mean differences between cases and controls in vascular risk factor exposures of interest by database and time prior to index date

**Supplementary Table 1: Vascular risk factor status at each measurement period by database**

| Risk factors | Case/ Control status | 10- 12 years | 8- 10 years | 6- 8 years | 4- 6 years | 2- 4 years | 0- 2 Years* |
|---|---|---|---|---|---|---|---|
| **AUH** | | | | | | | |
| Mean BMI (SD) | Case | | | | 26.03 (4.36) | 24.96 (4.74) | 24.27 (4.56) |
| | Control | | | | 25.40 (4.49) | 25.56 (4.46) | 25.38 (4.61) |
| Mean Total cholesterol (SD) | Case | 5.75 (1.10) | 5.44 (1.03) | 5.27 (1.07) | 5.11 (1.06) | 4.99 (1.09) | 4.86 (1.10) |
| | Control | 5.72 (1.04) | 5.46 (1.07) | 5.26 (1.08) | 5.12 (1.05) | 5.00 (1.05) | 4.87 (1.06) |
| **HSD** | | | | | | | |
| Risk factors | Case/ Control status | 10- 12 years | 8- 10 years | 6- 8 years | 4- 6 years | 2- 4 years | 0- 2 Years |
| Mean BMI (SD) | Case | 27.33 (4.59) | 27.59 (4.76) | 27.29 (4.72) | 26.98 (4.79) | 26.71 (4.87) | 26.40 (4.89) |
| | Control | 27.35 (4.39) | 27.46 (4.25) | 27.44 (4.25) | 27.27 (4.21) | 27.07 (4.25) | 26.90 (4.38) |
| Mean SBP (SD) | Case | 143.84 (15.78) | 142.95 (15.47) | 141.99 (15.28) | 140.72 (15.39) | 139.44 (15.38) | 137.22 (15.64) |
| | Control | 144.95 (15.49) | 144.11 (15.23) | 143.07 (15.26) | 141.95 (15.12) | 140.65 (14.93) | 139.03 (14.69) |
| Mean DBP (SD) | Case | 82.97 (7.70) | 82.17 (7.42) | 81.34 (7.40) | 80.55 (7.36) | 79.72 (7.31) | 78.83 (7.38) |
| | Control | 81.93 (7.56) | 81.48 (7.42) | 80.59 (7.30) | 79.89 (7.29) | 79.16 (7.45) | 78.24 (7.71) |
| Mean Total cholesterol (SD) | Case | 5.95 (0.98) | 5.85 (0.99) | 5.73 (1.01) | 5.63 (1.01) | 5.51 (1.02) | 5.39 (1.04) |
| | Control | 5.93 (1.02) | 5.83 (1.03) | 5.70 (1.05) | 5.59 (1.06) | 5.47 (1.08) | 5.31 (1.10) |
| **IPCI** | | | | | | | |
| Risk factors | Case/ Control status | 10- 12 years | 8- 10 years | 6- 8 years | 4- 6 years | 2- 4 years | 0- 2 Years |
| Mean BMI (SD) | Case | 27.78 (4.64) | 27.48 (4.62) | 27.26 (4.45) | 27.11 (4.37) | 26.80 (4.41) | 26.25 (4.54) |
| | Control | 28.01 (4.64) | 27.83 (4.74) | 27.62 (4.74) | 27.57 (4.63) | 27.54 (4.61) | 27.32 (4.54) |
| Mean SBP (SD) | Case | 150.35 (17.94) | 149.65 (17.80) | 147.98 (17.74) | 146.22 (17.24) | 144.34 (17.39) | 141.25 (17.75) |

For peer review only

| | | | | | | | |
|---|---|---|---|---|---|---|---|
| | Control | 150.86 (17.86) | 150.08 (17.25) | 148.63 (17.03) | 147.31 (16.94) | 145.88 (16.90) | 144.28 (16.89) |
| Mean DBP (SD) | Case | 83.68 (8.40) | 82.75 (8.39) | 81.41 (8.26) | 80.23 (8.53) | 79.02 (8.61) | 77.75 (8.81) |
| | Control | 84.18 (8.16) | 82.88 (8.28) | 81.59 (8.40) | 80.52 (8.41) | 79.47 (8.48) | 78.45 (8.48) |
| Mean Total cholesterol (SD) | Case | 5.70 (1.11) | 5.52 (1.11) | 5.34 (1.17) | 5.23 (1.16) | 5.12 (1.15) | 5.01 (1.15) |
| | Control | 5.73 (1.01) | 5.48 (1.13) | 5.33 (1.17) | 5.24 (1.19) | 5.18 (1.14) | 5.07 (1.12) |

| Risk factors | Case/ Control status | | | **SIDIAP** | | | |
|---|---|---|---|---|---|---|---|
| | | 10- 12 years | 8- 10 years | 6- 8 years | 4- 6 years | 2- 4 years | 0- 2 Years |
| Mean BMI (SD) | Case | | 29.10 (4.39) | 28.79 (4.47) | 28.49 (4.45) | 28.11 (4.49) | 27.54 (4.55) |
| | Control | | 29.74 (4.53) | 29.49 (4.54) | 29.23 (4.56) | 28.97 (4.61) | 28.67 (4.68) |
| Mean SBP (SD) | Case | | 137.73 (15.19) | 137.33 (14.54) | 136.95 (14.36) | 136.10 (14.18) | 134.19 (14.33) |
| | Control | | 138.56 (14.24) | 138.23 (14.08) | 137.73 (13.78) | 136.96 (13.43) | 135.90 (13.03) |
| Mean DBP (SD) | Case | | 76.49 (8.18) | 75.75 (7.88) | 75.02 (7.95) | 74.21 (8.00) | 73.15 (8.17) |
| | Control | | 77.12 (7.90) | 76.47 (7.91) | 75.51 (7.90) | 74.56 (7.98) | 73.49 (7.99) |
| Mean Total cholesterol (SD) | Case | | 5.39 (0.93) | 5.35 (0.93) | 5.30 (0.94) | 5.22 (0.96) | 5.10 (0.99) |
| | Control | | 5.43 (0.93) | 5.38 (0.93) | 5.32 (0.94) | 5.26 (0.97) | 5.16 (0.98) |

| Risk factors | Case/ Control status | | | **THIN** | | | |
|---|---|---|---|---|---|---|---|
| | | 10- 12 years | 8- 10 years | 6- 8 years | 4- 6 years | 2- 4 years | 0- 2 Years |
| Mean BMI (SD) | Case | 26.74 (4.77) | 26.66 (4.83) | 26.47 (4.87) | 26.2 (8.90) | 25.75 (5.03) | 24.95 (5.19) |
| | Control | 26.57 (4.49) | 26.57 (4.54) | 26.53 (4.60) | 26.41 (4.68) | 26.32 (4.75) | 26.18 (4.89) |
| Mean SBP (SD) | Case | 146.09 (19.46) | 145.11 (19.00) | 144.23 (18.32) | 142.98 (17.96) | 140.59 (17.84) | 136.75 (17.34) |
| | Control | 148.22 (18.33) | 147.47 (17.80) | 146.46 (17.44) | 145.04 (17.04) | 143.35 (16.74) | 141.29 (16.61) |
| Mean DBP (SD) | Case | 81.39 (9.69) | 80.36 (8.64) | 79.44 (9.37) | 78.47 (9.30) | 77.21 (9.28) | 75.72 (9.13) |
| | Control | 81.05 (8.88) | 80.00 (8.73) | 78.97 (8.69) | 77.93 (8.54) | 76.99 (8.43) | 75.93 (8.42) |
| Mean Total cholesterol (SD) | Case | 5.20 (1.52) | 5.07 (1.38) | 4.98 (1.30) | 4.89 (1.25) | 4.82 (1.22) | 4.75 (1.19) |

For peer review only

| Risk factors | Case/ Control status | 10- 12 years | 8- 10 years | 6- 8 years | 4- 6 years | 2- 4 years | 0- 2 Years |
|---|---|---|---|---|---|---|---|
| | Control | 5.32 (1.31) | 5.05 (1.31) | 4.90 (1.28) | 4.83 (1.24) | 4.79 (1.20) | 4.73 (1.18) |
| | | | | | **IMIM-UPF** | | |
| Mean BMI (SD) | Case | | 28.69 (4.50) | 29.33 (5.13) | 29.01 (4.92) | 27.06 (4.43) | 26.88 (4.63) |
| | Control | | 30.35 (4.70) | 29.28 (4.86) | 28.85 (4.49) | 28.42 (4.58) | 27.92 (4.51) |
| Mean Total cholesterol (SD) | Case | 5.31 (1.09) | 5.26 (1.09) | 5.14 (1.15) | 5.01 (1.17) | 4.84 (1.15) | 4.27 (1.08) |
| | Control | 5.50 (1.07) | 5.35 (1.10) | 5.18 (1.12) | 5.01 (1.12) | 4.86 (1.13) | 4.71 (1.12) |

*Time periods (years prior to index date)

For peer review only

**Supplementary Table 2: Proportions of patients with missing data for each vascular risk factor in each database**

| All databases | | 10- 12 | 8- 10 | 6- 8 | 4- 6 | 2- 4 | 0-2* |
|---|---|---|---|---|---|---|---|
| BMI | Cases | 91% | 85% | 77% | 69% | 61% | 52% |
| | Control | 87% | 81% | 75% | 67% | 60% | 56% |
| SBP | Cases | 78% | 70% | 59% | 47% | 34% | 19% |
| | Control | 70% | 62% | 51% | 40% | 29% | 22% |
| DBP | Cases | 78% | 70% | 59% | 47% | 34% | 19% |
| | Control | 70% | 62% | 51% | 40% | 29% | 22% |
| Total Cholesterol | Cases | 92% | 85% | 75% | 64% | 53% | 39% |
| | Control | 89% | 82% | 72% | 61% | 51% | 43% |

| AUH | | 10- 12 | 8- 10 | 6- 8 | 4- 6 | 2- 4 | 0-2* |
|---|---|---|---|---|---|---|---|
| BMI | Cases | | | | 99% | 87% | 50% |
| | Control | | | | 98% | 84% | 62% |
| Total Cholesterol | Cases | 78% | 65% | 55% | 44% | 38% | 23% |
| | Control | 79% | 68% | 55% | 44% | 35% | 29% |

| HSD | | 10- 12 | 8- 10 | 6- 8 | 4- 6 | 2- 4 | 0-2* |
|---|---|---|---|---|---|---|---|
| BMI | Cases | 94% | 91% | 88% | 85% | 82% | 78% |
| | Control | 94% | 91% | 88% | 85% | 82% | 79% |
| SBP | Cases | 79% | 71% | 64% | 57% | 52% | 47% |
| | Control | 80% | 72% | 64% | 57% | 52% | 49% |
| DBP | Cases | 79% | 71% | 63% | 57% | 52% | 47% |
| | Control | 80% | 72% | 64% | 57% | 52% | 49% |
| Total Cholesterol | Cases | 83% | 77% | 70% | 64% | 60% | 54% |
| | Control | 82% | 76% | 69% | 63% | 57% | 53% |

| IPCI | | 10- 12 | 8- 10 | 6- 8 | 4- 6 | 2- 4 | 0-2* |
|---|---|---|---|---|---|---|---|
| BMI | Cases | 93% | 88% | 83% | 76% | 72% | 67% |
| | Control | 92% | 88% | 82% | 75% | 70% | 65% |
| SBP | Cases | 59% | 53% | 45% | 36% | 29% | 18% |
| | Control | 54% | 48% | 41% | 33% | 27% | 21% |
| DBP | Cases | 60% | 53% | 45% | 36% | 29% | 18% |
| | Control | 55% | 48% | 41% | 41% | 27% | 21% |
| Total Cholesterol | Cases | 83% | 77% | 70% | 63% | 58% | 55% |
| | Control | 83% | 77% | 67% | 48% | 53% | 50% |

For peer review only

| SIDIAP | | 10- 12 | 8- 10 | 6- 8 | 4- 6 | 2- 4 | 0-2* |
|---|---|---|---|---|---|---|---|
| BMI | Cases | | 93% | 80% | 67% | 54% | 45% |
| | Control | | 93% | 82% | 68% | 55% | 46% |
| SBP | Cases | | 90% | 74% | 54% | 35% | 20% |
| | Control | | 90% | 75% | 56% | 36% | 24% |
| DBP | Cases | | 90% | 74% | 54% | 54% | 20% |
| | Control | | 90% | 75% | 56% | 99% | 24% |
| Total Cholesterol | Cases | | 92% | 77% | 60% | 41% | 24% |
| | Control | | 93% | 78% | 61% | 44% | 32% |

| THIN | | 10- 12 | 8- 10 | 6- 8 | 4- 6 | 2- 4 | 0-2* |
|---|---|---|---|---|---|---|---|
| BMI | Cases | 83% | 77% | 72% | 67% | 60% | 52% |
| | Control | 74% | 69% | 65% | 60% | 58% | 57% |
| SBP | Cases | 68% | 60% | 52% | 44% | 34% | 16% |
| | Control | 50% | 42% | 35% | 28% | 22% | 18% |
| DBP | Cases | 68% | 60% | 52% | 44% | 34% | 16% |
| | Control | 50% | 42% | 35% | 28% | 22% | 18% |
| Total Cholesterol | Cases | 88% | 82% | 75% | 69% | 61% | 48% |
| | Control | 83% | 76% | 68% | 61% | 55% | 50% |

| IMIM-UPF | | 10- 12 | 8- 10 | 6- 8 | 4- 6 | 2- 4 | 0-2* |
|---|---|---|---|---|---|---|---|
| BMI | Cases | | 99% | 99% | 98% | 98% | 94% |
| | Control | | 99% | 99% | 98% | 97% | 95% |
| Total Cholesterol | Cases | 86% | 81% | 76% | 70% | 64% | 31% |
| | Control | 87% | 83% | 79% | 75% | 71% | 68% |

*Time periods (years prior to index date)

For peer review only

**Supplementary Table 3: Meta-analysed mean number of missing data points* (95% confidence intervals)**

| | BMI | | SBP | | DBP | | Total cholesterol | |
|---|---|---|---|---|---|---|---|---|
| **Variable** | **Control** | **Case** | **Control** | **Case** | **Control** | **Case** | **Control** | **Case** |
| **Age at index date** | | | | | | | | |
| Under 60 | 2.75 (1.74, 3.76) | 2.54 (1.13, 3.95) | 2.78 (1.77, 3.79) | 2.77 (1.68, 3.86) | 2.78 (1.77, 3.79) | 2.77 (1.68, 3.86) | 2.67 (1.42, 3.92) | 2.58 (1.46, 3.70) |
| 60- 69 | 2.81 (1.74, 3.88) | 2.76 (1.28, 4.24) | 2.75 (1.77, 3.73) | 2.73 (1.50, 3.96) | 2.75 (1.77, 3.73) | 2.73 (1.50, 3.96) | 2.89 (1.43, 4.35) | 2.29 (0.84, 3.74) |
| 70- 79 | 2.89 (1.83, 3.95) | 2.84 (1.02, 4.66) | 2.81 (1.63, 3.99) | 2.85 (1.56, 4.14) | 2.81 (1.63, 3.99) | 2.85 (1.56, 4.14) | 2.28 (1.27, 3.29) | 2.44 (1.15, 3.73) |
| 80- 89 | 2.81 (1.25, 4.37) | 2.86 (1.3, 4.42) | 2.17 (0.85, 3.49) | 2.67 (1.51, 3.83) | 2.17 (0.85, 3.49) | 2.67 (1.51, 3.83) | 2.76 (1.55, 3.97) | 2.54 (1.22, 3.86) |
| 90 and over | 2.54 (1.13, 3.95) | 2.65 (1.16, 4.14) | 2.71 (1.26, 4.16) | 2.45 (1.16, 3.74) | 2.71 (1.26, 4.16) | 2.45 (1.16, 3.74) | 2.44 (1.10, 3.78) | 2.77 (1.42, 4.12) |
| **Gender** | | | | | | | | |
| Male | 2.78 (1.19, 4.37) | 2.71 (1.08, 4.34) | 2.60 (1.51, 3.69) | 2.74 (1.69, 3.79) | 2.60 (1.51, 3.69) | 2.74 (1.69, 3.79) | 2.24 (1.23, 3.25) | 2.56 (1.47, 3.65) |
| Female | 2.71 (1.28, 4.14) | 2.75 (1.17, 4.33) | 2.69 (1.37, 4.01) | 2.64 (1.38, 3.90) | 2.69 (1.37, 4.01) | 2.64 (1.38, 3.90) | 2.32 (1.09, 3.55) | 2.48 (1.34, 3.62) |
| **Co-morbidities** | | | | | | | | |
| AMI | 2.55 (1.47, 3.63) | 2.63 (1.38, 3.88) | 3.01 (1.94, 4.08) | 2.97 (1.85, 4.09) | 3.01 (1.94, 4.08) | 2.97 (1.85, 4.09) | 2.12 (0.88, 3.36) | 2.10 (0.64, 3.56) |
| DM | 2.76 (1.62, 3.90) | 2.71 (1.35, 4.07) | 2.70 (1.45, 3.95) | 2.75 (1.63, 3.87) | 2.70 (1.45, 3.95) | 2.75 (1.63, 3.87) | 2.07 (0.62, 3.52) | 2.13 (0.77, 3.49) |

For peer review only

| | | | | | | | | |
|---|---|---|---|---|---|---|---|---|
| Stroke | 2.82 (1.59, 4.05) | 2.78 (1.32, 4.24) | 2.81 (1.36, 4.26) | 2.72 (1.49, 3.95) | 2.81 (1.36, 4.26) | 2.72 (1.49, 3.95) | 2.87 (1.01, 4.73) | 2.39 (0.72, 4.06) |
| Hypertension | 2.12 (1.11, 3.13) | 2.05 (0.80, 3.30) | 2.43 (1.32, 3.54) | 2.39 (1.38, 3.40) | 2.43 (1.32, 3.54) | 2.39 (1.38, 3.40) | 2.19 (0.78, 3.60) | 2.14 (0.55, 3.73) |
| Total sample | 2.75 | 2.73 | 2.67 | 2.66 | 2.67 | 2.66 | 2.30 | 2.50 |

*From a denominator of 6 data points apart from the following: UPF-BMI 5 data points; AUH-BMI 3 data points; SIDIAP variables 5 data points

AMI: acute myocardial infarction; DM: type 2 diabetes mellitus

For peer review only

**STROBE 2007 (v4) Statement—Checklist of items that should be included in reports of *case-control studies***

| **Section/Topic** | **Item #** | **Recommendation** | **Reported on page #** |
|---|---|---|---|
| **Title and abstract** | 1 | (*a*) Indicate the study's design with a commonly used term in the title or the abstract | 5 |
| | | (*b*) Provide in the abstract an informative and balanced summary of what was done and what was found | 5- 6 |
| **Introduction** | | | |
| Background/rationale | 2 | Explain the scientific background and rationale for the investigation being reported | 8 |
| Objectives | 3 | State specific objectives, including any prespecified hypotheses | 8- 9 |
| **Methods** | | | |
| Study design | 4 | Present key elements of study design early in the paper | 10 |
| Setting | 5 | Describe the setting, locations, and relevant dates, including periods of recruitment, exposure, follow-up, and data collection | 10- 12 |
| Participants | 6 | *(*a) Give the eligibility criteria, and the sources and methods of case ascertainment and control selection. Give the rationale for the choice of cases and controls | 10- 11 |
| | | (*b*) For matched studies, give matching criteria and the number of controls per case | 10- 11 |
| Variables | 7 | Clearly define all outcomes, exposures, predictors, potential confounders, and effect modifiers. Give diagnostic criteria, if applicable | 11 |
| Data sources/ measurement | 8* | For each variable of interest, give sources of data and details of methods of assessment (measurement). Describe comparability of assessment methods if there is more than one group | 11 |
| Bias | 9 | Describe any efforts to address potential sources of bias | 18, Supplementary table 2 |
| Study size | 10 | Explain how the study size was arrived at | 10 |
| Quantitative variables | 11 | Explain how quantitative variables were handled in the analyses. If applicable, describe which groupings were chosen and why | 12 |
| Statistical methods | 12 | (*a*) Describe all statistical methods, including those used to control for confounding | |
| | | (*b*) Describe any methods used to examine subgroups and interactions | 12 |
| | | (*c*) Explain how missing data were addressed | 12 and Supplementary table 2 |

For peer review only

| | | | |
|---|---|---|---|
| | | (*d*) If applicable, explain how matching of cases and controls was addressed | 10- 11 |
| | | (*e*) Describe any sensitivity analyses | |
| **Results** | | | |
| Participants | 13* | (a) Report numbers of individuals at each stage of study—eg numbers potentially eligible, examined for eligibility, confirmed eligible, included in the study, completing follow-up, and analysed | 13- 14, supplementary table 2 and 3 |
| | | (b) Give reasons for non-participation at each stage | 13- 14, supplementary table 2 and 3 |
| | | (c) Consider use of a flow diagram | |
| Descriptive data | 14* | (a) Give characteristics of study participants (eg demographic, clinical, social) and information on exposures and potential confounders | Table 1 in page 21 |
| | | (b) Indicate number of participants with missing data for each variable of interest | supplementary table 2 |
| Outcome data | 15* | Report numbers in each exposure category, or summary measures of exposure | Table 1 and table 2 in page 21 and 22 and Supplementary Table 1 |
| Main results | 16 | (*a*) Give unadjusted estimates and, if applicable, confounder-adjusted estimates and their precision (eg, 95% confidence interval). Make clear which confounders were adjusted for and why they were included | Table 2 and table 3 in page 22 and 23 |
| | | (*b*) Report category boundaries when continuous variables were categorized | Page 11 |
| | | (*c*) If relevant, consider translating estimates of relative risk into absolute risk for a meaningful time period | |
| Other analyses | 17 | Report other analyses done—eg analyses of subgroups and interactions, and sensitivity analyses | Table 3 for subgroup analysis in page 23; |
| **Discussion** | | | |
| Key results | 18 | Summarise key results with reference to study objectives | Page 15 |
| Limitations | 19 | Discuss limitations of the study, taking into account sources of potential bias or imprecision. | Page 17-18 |

For peer review only

| | | | |
|---|---|---|---|
| | | Discuss both direction and magnitude of any potential bias | |
| Interpretation | 20 | Give a cautious overall interpretation of results considering objectives, limitations, multiplicity of analyses, results from similar studies, and other relevant evidence | Page 18- 19 |
| Generalisability | 21 | Discuss the generalisability (external validity) of the study results | Page 18- 19 |
| **Other information** | | | |
| Funding | 22 | Give the source of funding and the role of the funders for the present study and, if applicable, for the original study on which the present article is based | Page 2 |

*Give information separately for cases and controls in case-control studies and, if applicable, for exposed and unexposed groups in cohort and cross-sectional studies.

**Note:** An Explanation and Elaboration article discusses each checklist item and gives methodological background and published examples of transparent reporting. The STROBE checklist is best used in conjunction with this article (freely available on the Web sites of PLoS Medicine at http://www.plosmedicine.org/, Annals of Internal Medicine at http://www.annals.org/, and Epidemiology at http://www.epidem.com/). Information on the STROBE Initiative is available at www.strobe-statement.org.

For peer review only
